# Computational chemistry methods based on MNDO and tools for improving their accuracy

**DOI:** 10.1007/s00894-026-06653-3

**Published:** 2026-03-13

**Authors:** James J. P. Stewart

**Affiliations:** Stewart Computational Chemistry, 15210 Paddington Circle, Colorado Springs, CO 80921 USA

**Keywords:** Semiempirical method development, Causes of error, MNDO, Development of PM6-ORG, Modeling proteins, Applicability

## Abstract

**Context:**

Semiempirical computational methods based on the MNDO approximation have a long history. Since its release in 1977, the original MNDO method has evolved steadily, passing through several forms (AM1, PM3, etc.) up to the present. Each new form represented an improvement, and it is anticipated that the evolution will continue for many more years.

**Methods:**

A summary of the history of MNDO-based methods will be given, followed by a description of the mechanisms involved in developing some of the current methods. To assist in this, a set of files illustrating the steps and procedures involved is provided in Supporting Material; hopefully these will be useful in facilitating the development of future methods.

**Supplementary Information:**

The online version contains supplementary material available at 10.1007/s00894-026-06653-3.

## Introduction

Semiempirical (SE) methods have been proven useful in modeling chemical behavior. Despite their apparent complexity, SE methods have only three possible sources of error, which implies that if these errors were to be eliminated, SE methods would be completely accurate. Given that they are not, the origin of the errors needs to be examined and mechanisms developed to eliminate them. These three sources are (1) faults and limitations in the theoretical framework, (2) incomplete parameter optimization, and (3) faults in reference data. Each of these will now be examined.

### Evolution of the theoretical framework

The genesis of modern SE methods can be dated as June 1, 1977, when Dewar and Thiel published their seminal article on the MNDO method [1]. This method was parameterized using reference data for 32 small compounds restricted to the elements hydrogen, carbon, nitrogen, and oxygen. Values for a total of 22 adjustable parameters were optimized, and the predictive power of the new method was determined by a survey of 138 molecules [2]. MNDO immediately became the focus of enthusiastic research efforts and was quickly extended to several other elements.

In 1983, the MNDO method was incorporated into a modeling program, MOPAC [3]. Since then, both it and a parameter optimization program, PARAM, have been under continuous development and can now be regarded as mature and stable platforms for the tasks they were designed to perform.

Over the years, several changes were made to the theoretical framework in MOPAC, some of the more important being:

The extension of the MNDO method to modeling systems containing hypervalent main-group elements or transition metals had been impractical due to the absence of d-orbitals. This limitation was corrected in 1992 by Thiel and Voityuk [4] and was applied successfully [5] four years later to modeling both types of system.

The COSMO solvation model [6] was developed in 1993, and its first implementation was in MOPAC. This allowed systems in solution, particularly water, to be modeled, which opened the ability to use SE methods to model biochemical systems. Several other solvation models were also developed around that time. For a review, see Cramer and Truhlar [7].

In 1996, an alternative to the use of matrix algebra to solve the self-consistent field equations was developed. This technique [8], named MOZYME, used localized molecular orbitals, which allowed all quantum effects to be handled locally. An unavoidable consequence of using the MOZYME technique was that the resulting molecular orbitals were not eigenfunctions, but because eigenfunctions are not observables, and observables are the only quantities of interest in chemistry, this limitation should not be regarded as important. A similar situation occurs in pseudodiagonalization [9, 10]. In this case, the starting M.O.s are eigenfunctions, unlike those in MOZYME, where the starting M.O.s are derived from the Lewis structure and involve only one or two atoms. The MOZYME technique has allowed large systems, up to about 10,000 atoms, to be modeled.

In 2012, two errors relating to non-covalent interactions were corrected. Until then, the effects of the London dispersion force had not been included, and the strength of the non-covalent hydrogen bond had been underestimated. To address these faults, Řezáč and Hobza used Grimme’s original dispersion correction [11] together with a hydrogen bond correction to develop the “D3H4” [12] correction; which gave good results for the intermolecular interactions in even very large systems, such as Grimme’s S12L test set [13]. In order to comply with the Hellmann-Feynman theorem [14, 15], corrections of this type must not involve the spatial distribution of the electrons. In particular, they must not involve atomic partial charges. Two tests were done to confirm that the Hellman-Feynman theorem was being complied with: in one, examination of “D3” and “H4” correction equations confirmed that no terms relating to the electron distribution were present, while, in the other, it was confirmed that the square of the derivatives of the heats of formation were all at a minimum when the heats of formation were at a minimum.

Finally, also in 2012, an error was found in the point-charge repulsion in the NDDO approximation [16]. This error, although for all practical purposes unimportant in molecular systems, would become infinitely large if rigorously applied to solids. Correcting the error was simple and gave rise to a new method, PM7 [17], and a reduction of 60% in the average unsigned error in heats of formation (ΔH_f_) at standard temperature and pressure for organic solids.

One approach that was both more theoretically sound than the original MNDO method and more accurate than the then-available SE methods resulted in Thiel’s OMx orthogonalization-corrected methods [18]. Evaluating the gradients posed some problems mainly relating to the computational effort involved; for that reason, the OMx approach was not pursued further.

### Parameter optimization

Modeling a chemical system using SE methods requires significantly less computational time than that required when ab initio methods are used. This increase in speed is a consequence of replacing various computationally intensive steps with adjustable parameters. Optimizing the values of these parameters involves minimizing an error function defined as the sum of squares of the weighted differences between calculated and reference data.

Several very different types of reference data are needed in parameter optimization, the main types being standard ΔH_f_, molecular geometry, ionization potential, and molecular dipole moment. An essential first step in converting reference data into data suitable for the optimization process is to render them dimensionless, which is achieved by multiplying the data by appropriate weighting factors.

#### The parameter Hessian

At the core of parameter optimization is the parameter Hessian matrix [17], the elements of which are the second-order partial derivatives of the error function with respect to the various adjustable parameters. Given that the objective of parameter optimization is to minimize a sum of squares, an estimate of this sum at points near to the current position on the parameter hypersurface can be obtained by using the fact that this surface is parabolic in all directions.

By diagonalizing the Hessian, the resulting eigenvectors give the orthogonal directions in the parameter hyperspace and, more importantly, the associated eigenvalues indicate the rate of curvature in the relevant directions. All the eigenvalues are, by definition, positive, but if any of the eigenvalues are very small or even zero, then motion along the associated eigenvectors would have little or no effect on the error function. When this situation occurs, then, by definition, the parameter set would be ill-defined, a severe fault that could only be corrected by either reducing the size of the parameter space or, more easily, by adding reference data for which the calculated value would be influenced by motion along the low-lying eigenvectors.

A vivid example of this is provided by ionization potentials. If no ionization potentials or ΔH_f_ of any ionized species were present in the reference data, then at least one parameter eigenvalue would be precisely zero, and a constant could then be added to all the one-center one-electron energies, *U*_*ss*_, *U*_*pp*_, and *U*_*dd*_, without affecting any calculated ΔH_f_, dipole moments, or geometries. However, as soon as at least one such reference datum is present, the relevant eigenvalue becomes non-zero, and all the one-center one-electron energies would become defined.

Access to the Hessian can thus give information on the type of reference data that would be necessary to obtain a well-defined parameter hyperspace. Conversely, if two or more reference data were similar, such as the ΔH_f_ of butane and pentane, then their contributions to the hyperspace would also be similar, and therefore, by definition, deleting one or the other of these reference data would have little effect on the low-lying eigenvectors. This behavior would help indicate when reference data could be removed without compromising the Hessian.

The parameter Hessian is used in Newton-Raphson function minimization, where an improved value of the error function can be obtained by simply calculating successive points on the parameter hypersurface. If this were to be done here, the frequent function evaluation would be extremely computationally demanding. Fortunately, use can be made of the parabolic nature of the hypersurface to predict the value of the error function in the region near to the current point. By using the Hessian to predict the error function, larger steps can be taken before the accumulated errors would require an explicit recalculation of the error function and its derivatives. In practice, up to 2000 steps based on the Hessian could be done before an explicit recalculation became necessary.

Over the past 40 years, the various components of PARAM have undergone steady changes to improve their rigor and efficiency and, in general, to make it easier to use.

### Reference data

Optimizing parameter values requires a large amount of accurate reference data. Unfortunately, the quality and availability of suitable experimentally determined reference data for standard ΔH_f_ is poor. The dearth of reference data can be understood by the fact that only small molecules can be used in parameterization, and for standard ΔH_f_ these have to be gas-phase. Of the first 83 elements, only about 20 have suitable gas-phase compounds, which means that almost all the metals, i.e., most of the periodic table, are excluded. This, then, currently represents the greatest obstacle in developing SE methods. One potentially promising alternative to using experimental ΔH_f_ would be to use theoretically predicted values, such as those predicted [19] using ab initio density function theory.

### Derived reference data

Some reference data are of great importance, but cannot be represented by simple heats of formation. Examples of this type are heats of reaction and non-covalent interaction energies. Reference data for these can be expressed in terms of an energy and a chemical system. Thus, the heat of reaction, ΔH(R), to form a molecular system “B” from reactants “A” can be expressed as ΔH(R) = ΔH_f_(B) - ΔH_f_(A), where ΔH_f_(B) and ΔH_f_(A) are the heats for formation of species A and B, respectively. Using this convention, the derived reference value of ΔH(R) can then be used as a normal reference datum.

For many derived reference data, particularly those involving non-covalent interactions, the energies involved, i.e., the ΔH(R), are small, but are of greater importance than the heats of formation. To allow for this increased importance, the weighting factor can be adjusted as necessary.

#### Reported experimentally determined ΔH_f_

By definition, physical science involves the study of the properties of the real world. Also, by definition, reality can be regarded as accurate, so the standard ΔH_f_ of chemicals should, again by definition, be regarded as a property of those chemicals. Unfortunately, these definitions cannot be used to imply that reported experimental ΔH_f_ is accurate.

The National Institute of Standards and Technology (NIST) Chemistry WebBook [20] has proven to be a valuable source of reference data both for method development and for surveys in which predicted results were compared with reference data. Indeed, for many decades, the high quality of the NIST data has allowed the accuracy of new SE methods to be determined. For most systems, the agreement was good, and in most instances where the differences were large, there was unambiguous evidence that the discrepancy was being caused by the limited accuracy of the semiempirical method. However, in 2004, strong evidence was presented [21] that 20 of the entries in the WebBook were inaccurate. For all these entries, the semiempirical calculated ΔH_f_ was significantly different from the reported value, and further examination did not reveal any cause for the discrepancies that could be attributed to the computational model. In addition, in all cases, the reported anomalous ΔH_f_ were found to be inconsistent with those of closely related compounds. For most of these, a review of the original publications did not provide a specific reason for the anomaly, but in a few cases a hitherto unnoticed error was identified. Two egregious examples illustrating this are:

- In one case, an error was caused by the use of an incorrect chemical formula: in the procedure for deriving the ΔH_f_ of 2,6,6-trimethyl-2-cyclohexen-1-one, the formula used [22] was C_9_H_13_O instead of the correct C_9_H_14_O. When the correct formula was used, the resulting ΔH_f_ was in good agreement with both the calculated value and those of closely related compounds.
- In another, a typographical error in the original article [23] resulted in the reported ΔH_f_ for o-tert-butyl-p-cresol being 207.0 kJ mol^−1^, this differing by 416.2 kJ mol^−1^ from the calculated value of −209.2 kJ mol^−1^. The reported value was also inconsistent with the ΔH_f_ of related compounds such as 2-isopropyl-m-cresol, at −172 kJ.mol^−1^. Clearly a minus sign had been inadvertently omitted from the experimental value. When it was inserted, the difference between the calculated and the experimentally determined value dropped to 2.2 kJ mol^−1^.

After reviewing the evidence of errors in experimental work, NIST made changes to the WebBook to make corrections or, in some cases, delete irremediable experimental data entries.

#### Parameterization set and Survey set

A large amount of reference data is used in parameter optimization, and this same data is also used in surveying the resulting method to determine its accuracy. The decision to adopt this strategy was justified by the large ratio of the number of reference data used in parameterizing to the number of parameters to be optimized, typically a factor of 10 or more, and by the large ratio of the number of reference data used in the surveys to the number used in the parameter optimization. These ratios effectively negate any bias from the parameter optimization affecting the survey results.

## Current method development

### Software

At the present time, the theoretical framework in PM6-ORG [24], a method optimized for modeling proteins and containing the correction to the NDDO point-charge repulsion, is sufficiently flexible that the framework currently provides a sufficiently robust basis for existing applications, although the possibility exists that further specialized refinements may emerge. The parameter optimization is also stable and is flexible enough to allow several hundred parameters to be optimized simultaneously, using several thousand reference data. Once a working parameterization data set has been constructed, making further changes is easy.

Various partial optimizations based on PM6 [25] have been reported that focus on intermolecular interactions. In these methods, the reference data consist of high-level ab initio predictions of non-covalent interaction energies and geometries of the various pairs of species, and the parameters optimized were those involved in the terms relating to these interactions.

Although the resulting methods reproduced intermolecular properties with a much-improved accuracy, the presence of these additional terms introduced errors in the calculated ΔH_f_: thus, for example, the average unsigned errors in ΔH_f_ for PM6 and PM6-D3H4 [12] in a survey of 2176 data increased from 6.4 to 13.2 kcal mol^−1^. This had the effect of reducing the range of applicability of the resulting methods. Conversely, if all parameters are allowed to optimize, the change in average unsigned errors would become much smaller. This can be illustrated by PM6-ORG, which was deliberately biased towards modeling intermolecular properties of the type found in proteins. As a result of all parameters being optimized, instead of only those in the D3H4 terms, the average unsigned error in ΔH_f_ decreased to 7.2 kcal mol^−1^, less than 1 kcal mol^−1^ larger than that of the parent PM6 method. The advantages of global optimization and the capability of the current PARAM mean that the current efficiency of PARAM makes full global optimization a highly accessible and recommended standard for future method development.

### Reference data

Several very different types of reference data are needed in parameterizing new methods. The original method, MNDO, used only one type: data on small molecules. While this type is still in use, in order to increase the flexibility of SE methods, the range of elements and types of species has had to be increased considerably.

#### Sources

The WebBook has proven to be a valuable source of reference data on ΔH_f_, and for geometries, the Cambridge Crystallographic Data Center [26] and the results of high-level ab initio calculations are ideal. High-level ab initio calculations have also been a valuable source of data on I.P.s and dipoles.

In recent years, the importance of non-covalent interaction energies has increased, and in response to this, a second family of specialized reference data, consisting mainly of entries from the Non-Covalent Interactions Atlas project [27], has been added. The Atlas contains several collections of benchmark interaction energies that have been invaluable as reference data for parameterizing and for surveying non-covalent bonding energies and geometries. Specific collections of particular value here include the R739 collection [28] of repulsive interactions, the IHB100 collection [29] of ionic hydrogen bonds in organic molecules, and the HB375 collection [29] of hydrogen bonds. The result of reparameterization was the PM6-ORG method in which the average unsigned error in energies for 688 pairs of non-covalently interacting species dropped by 5% relative to PM6-D3H4 and, more importantly, the kurtosis in the repulsive interactions error distribution dropped from 12.6 to −0.1.

### Determining accuracy

For obvious reasons, determining the accuracy of a SE method is essential. To assist in quantifying this, a few small utility programs have been developed that construct tables of statistics for unsigned errors in ΔH_f_, dipole moments, I.P.’s, bond-lengths, and angles that allow quantitative comparisons to be made between methods. These tools have been very useful in guiding the development of each new method.

#### Proteins

Defining accuracy metrics for proteins presents an unusual challenge. Individual sections of protein chains can be regarded as simple organic species. As such, the accuracy of prediction of properties of small organic compounds, which has just been covered, is applicable. More important, however, are the inter- and intra-chain interactions. These, particularly the shorter non-covalent interactions, are of great experimental interest.

A popular and useful measure of the quality of a protein’s structure is provided by the validation program Molprobity [30]. In that, “clashes” give a measure of individual steric overlaps, and “clashscores” give a single measure of the quality of a protein structure. A second metric, useful in computational modeling, would be to compare the calculated volume of a protein with that of the reported experimental structure. This metric is important in that if a protein structure were to be opened out so that the non-covalent interaction distances increased, then the clashscore would automatically decrease. By monitoring the volumetric difference, the potential risk of this artifact distorting the significance of clashscores can be avoided. Perhaps fortunately, in practice all calculated volumes were slightly smaller than those experimentally determined, so this hazard is currently not very important.

Average clashscores for a test set [24] of 21 proteins were 4.7 for PM6-ORG and 7.5 for the experimental PDB files.

## Modeling

Several very different types of systems can be modeled, of which the most important types are the following:

### Proteins

There are two problems that are specific to large, complex systems such as proteins. Their great size makes modeling difficult because of the large computational effort involved, and achieving the high precision that is essential when working with relative ΔH_f_. In general, biochemical processes involve very small heats of reaction, so, to be of predictive use, computed energies need to have a precision of better than about 1.0 to 1.5 kcal mol^−1^. Simply put, achieving this precision is practically impossible in systems that contain many thousands of atoms.

Both these problems can be solved by reducing the size of the system to only the region of interest. For convenience, this region, plus any small molecules needed, will be referred to as a Globule [31, 32]. Deciding how large the Globule needs to be is not difficult: if atoms at the periphery do not move substantially when a ligand is docked in the binding site, then all atoms further out can be ignored.

Determining if SE methods can provide information useful in protein research would involve designing and running tests to establish their validity. Four such tests will now be described.

#### Ability to model biochemical processes

The protease chymotrypsin has been extensively studied both experimentally and by computational chemistry methods and thus provides a good system for testing the ability of a method for modeling enzymes. All six intermediates and five of the transition states in the catalytic cycle were identified and refined, and the nature and role of the catalytic triad and the oxyanion hole were investigated [33]. All results were in accord with the accepted hydrolysis mechanism. Therefore, the ability to model correctly at least one catalytic cycle was confirmed.

The PM7 method was used for this and the following two tests. During this work, the limitations of PM7 for modeling intermolecular interactions became evident, and these limitations provided the motivation for developing the significantly more accurate PM6-ORG method.

#### Investigating mechanisms

Another test involved investigating the mechanism of the human nucleotide pool sanitizing enzyme MTH1. The Great Oxygenation Event occurred about 2.4 billion years ago, at which time the Earth’s atmosphere changed from being reducing to being oxidizing, and free oxygen molecules became available. These were poisonous in that they oxidized nucleotide triphosphates, the building blocks of DNA, and when these oxidized nucleotides became incorporated in DNA, the results were harmful.

Over time, enzymes that could selectively hydrolyze nucleotide triphosphates evolved. These were unusual in that they had to discriminate between normal and oxidized nucleotides and then hydrolyze the triphosphate to give a nucleotide phosphate and a pyrophosphate ion. This reaction had to be highly specific; otherwise, either the essential triphosphate would also be destroyed, or the oxidized triphosphate would not be destroyed, and the resulting DNA would be damaged. Because the mechanism involved in discriminating between the two nucleotides was not yet known, although a conjecture had been made [34], this system was selected for a test to see if any useful predictions could be made.

Modeling the binding of both normal and oxidized nucleotide triphosphates to MTH1 allowed an inspection [35] of the various phenomena involved, which consisted of the docking stabilization energy, about 88 kcal mol^−1^, and the difference in binding energies of the two substrates, the oxidized form being the more stable by about 0.85 kcal mol^−1^. Further inspection of the environment of the substrates provided a plausible explanation for this result.

One result of this investigation was an explanation for the origin of the enzymes’ specificity. Another result was the inescapable need for high precision in all modeling operations, the specificity being due to the minute difference in binding energies, here less than 0.9 kcal mol^−1^, a level of precision that had not hitherto been anticipated.

#### Detecting errors

In 1999, Luecke et al., published [36] the structure of bacteriorhodopsin, a photon-activated proton pump comprised of a seven-transmembrane alpha-helix protein and a retinal Schiff base. Among the several lipid chains entrained in the crystal, one, SQU-701, was tentatively identified in the original article as squalene, a polyunsaturated hydrocarbon, but this moiety was referred to in the PDB file 1C3W as the saturated hydrocarbon 2, 10, 23-trimethyl-tetracosane, and the pattern of carbon atoms in residue 701 matched that name.

The number and positions of hydrogen atoms were not reported in the PDB file, but from the bond length alternation in SQU-701, the hydrogenation option in MOPAC assigned hydrogen atoms in the pattern in squalene. When the geometry of 1C3W was optimized, the bond-length alternation in SQU-701 was, as expected, a match for squalene.

In the 1C3W Full Validation Report, all six bond lengths outliers in SQU corresponded to the double bonds if SQU was indeed squalene and not to the single bonds in the saturated 2, 10, 23-trimethyl-tetracosane reported in the PDB.

Obviously no definitive conclusion can be made from these facts as to whether the lipid was squalene or 2, 10, 23-trimethyl-tetracosane, a hydrocarbon not mentioned in the article, but what can be said with certainty is that the modeling results raised flags indicating that there was an inconsistency between the article and the PDB entry.

#### Predicting ligand binding

The final test to establish the usefulness of SE methods for modeling proteins was to compare the calculated binding energy of various ligands to an enzyme of interest and then to correlate the results with the experimentally observed IC_50_ values.

The system chosen was of topical interest: the COVID-19 protease enzyme, PDB entry 7JUN [37], together with a series of ligands from the Collaborative Drug Discovery Database [38, 39] that shared a common backbone. By using closely related species, the ability to discriminate between the various ligands could be established.

As this was the first use of PM6-ORG to model a protein, preliminary tests, similar to the first of the three tests just described, were carried out; these confirmed that PM6-ORG would be able to reproduce known phenomena in the protease. A survey was then carried out to determine how accurately the binding energy correlated with the IC_50_. The coefficient of determination, *R*^2^, was found [31] to be about 0.7, a value sufficiently large to suggest that PM6-ORG might be of potential use in drug discovery.

#### Other phenomena

The version of MOPAC used in the modeling reported here would allow some other phenomena related to the electronic ground state of proteins, e.g., electrostatics, to be investigated. Some other phenomena, such as electronic spectra, would require an appropriate configuration interaction calculation to be performed. In principle, such calculations could be done, but the C.I. methods in MOPAC require the M.O.s to be eigenfunctions. Conversion of the localized molecular orbitals into eigenfunctions is a computationally intensive operation, and when proteins are involved, this operation would be very time-consuming. The alternative, using a C.I. method that does not require the M.O.s to be eigenfunctions, would likely also be very time-consuming. In short, at present, modeling excited state phenomena such as the electronic spectra of proteins is impractical.

### Transition states and reactions

Several different methods have been implemented in MOPAC for locating transition state geometries for chemical reactions. Some are very simple to generate. Transition geometries for narcissistic or degenerate rearrangement reactions can be obtained in one step by using symmetry to define the distance from the reacting atom to both the incoming and leaving atoms to be the same. The transition state is then obtained by energy minimization. Slightly more complicated are the almost narcissistic reactions, particularly SN2 reactions, where the incoming and leaving groups are different, e.g., F^−^ and Cl^−^. Transition states for these reactions can usually be located in two steps. The first step is the same as for the narcissistic reaction just described. The second step, however, involves gradient minimization instead of energy minimization. An alternative would be to map out the reaction path and select the geometry that has the highest ΔH_f_.

Of greater importance, however, are the complicated reactions in which several covalent bonds are made and broken. For such systems more sophisticated methods [40] such as LOCATE-TS and SADDLE are used, methods that are particularly useful when modeling enzyme-catalyzed reactions.

Once a transition state geometry has been obtained, mapping out the Intrinsic Reaction Coordinate (IRC) or Dynamic Reaction Coordinate (DRC) is not difficult [41].

### Solids

Computational modeling of crystalline solids is conventionally performed by sampling various points in the associated ***k***-space. Although this approach is efficient in that only one repeat unit needs to be used, it does require using weighting factors for each point, a process that, although straightforward for physicists, is less familiar to chemists. This could possibly help explain why modeling the chemical properties of solids has not progressed as fast as that for discrete chemical systems.

The approach used in MOPAC is to use the Large Unit Cell (LUC) model [42]. In this, either a single unit cell, if it is large enough, or several unit cells, for smaller unit cells, is used, the objective being to ensure that all quantum-mechanical interactions of every atom in the LUC interacting with all nearby atoms are taken into account. To achieve this, 26 other LUC images are constructed around the central LUC to give a 3 × 3 × 3 super-LUC. Outside this quantum-mechanical region, only simple electrostatic interactions are taken into account.

In conventional solid-state work, electrostatic interactions are taken into account using the Ewald summation [43–45]. This approach, although elegant, was found to be unsuitable when quantum effects were included. Specifically, quantum modeling also takes into account the same types of electrostatic interactions as are considered in the Ewald sum. This means that, if the Ewald sum method were to be used, then, to avoid double counting, the electrostatic contributions from the quantum region would have to be deleted. To avoid having to do this, an alternative approach based on the Madelung constant was developed [46].

By using the LUC model, the reciprocal space size is very small, and only the gamma point in the centre of the first Brillouin zone needs to be calculated; this allows solids to be modeled as easily as molecular systems. A second, incidental, advantage is that solids that have large unit cells can be modeled as easily as those with small unit cells, so, for example, buckminsterfullerene, C_60_, with a FCC unit cell (a = 14.15 Å) can be modeled as easily as diamond, C_2_.

A set of examples [47] of MOPAC datasets and images for various solids illustrates the strengths and weaknesses of using PM7 and PM6-D3H4 with this approach.

One property of solids that is of special interest is the effect of applied isotropic external pressure. By mapping the effect of different pressures, both positive and negative, the mechanical behavior of a solid under stress can be examined.

Various other phenomena involving the reciprocal space, such as vibrational (phonon) and electronic band structures, can be mapped and analyzed.

## Discussion

Unequivocally, attempting to develop a new SE method is a daunting process. The theoretical framework and optimization of its parameters took several years to complete before the original MNDO method could be published in 1977. Currently, because SE methods have to take into account many phenomena not addressed in MNDO, particularly those related to non-covalent interactions, modern SE methods are much more complicated and therefore involve many more parameters. In turn, optimizing the values of these parameters requires a very large amount of reference data, and the resulting parameter optimization process is correspondingly very computationally demanding.

A set of several utilities for manipulating reference data, preparing data sets for controlling optimization, and analyzing results has greatly simplified the process of method development. Because accuracy is of great importance, and as the largest source of errors in SE methods originates in the experimental reference data, it is highly likely that the greatest improvement can be made by using the results of high-level ab initio calculations as a source of more reliable reference data.

### Detecting errors in reference data

One potentially important application of a more reliable SE method would be to focus attention on potential errors in collections of experimentally determined reference data, such as the NIST WebBook and the PDB. These are, of their nature, susceptible to various errors—in measurement, in analysis, in transcription, etc.—and, unless attention is drawn to them, these errors might not be detected and therefore not corrected. Errors in SE predictions are, of their nature, different from those in the reference data collections, so when there is a large inconsistency between calculated and reported reference data, that can call attention to the cause of the error. The result would be to identify either a fault in the SE method or a fault in the experimental reference datum.

By detecting and analyzing the causes of these inconsistencies, the appropriate action can be taken to rectify them. Regardless of the cause, by focusing attention where it is needed, improvements could be made that would result in improved quality assurance.

### A problem involving modeling biochemical phenomena

As has been demonstrated here and in earlier articles, several biochemical mechanisms can be modeled with useful accuracy. However, many more simulations would be needed before much confidence can be placed in these models.

A somewhat surprising result of this work was that, by far, the most difficult step involved the construction and validation of a model of the system. In addition to using a good graphical package to visualize and edit the model, achieving the high precision needed required that great care had to be taken to ensure that the model was chemically realistic. Several times an initial model was found to be faulty, the fault only being discovered after computational modeling work had begun, at which point it was corrected. To complicate this picture, an unexpected problem was encountered involving a phenomenon known as “activity cliffs,” where the experimentally determined IC_50_ of a ligand was inconsistent with those of closely related ligands. In several cases, the computational model also predicted binding energy for the same ligand that was also inconsistent with the reported IC_50_. This discrepancy could not be resolved, despite almost a year of strenuous efforts, and, finally, a literature search revealed that the “activity cliff” effect [48, 49] was in fact a real phenomenon. This was a good, albeit frustrating, example of the Westheimer adage: “A couple of months in the laboratory can frequently save a couple of hours in the library.”

### Improving protein geometries

The quality of the overall structure that can be achieved using X-ray and neutron crystallography of the type used in generating structures such as the 7JUN model of the COVID-19 main protease enzyme, described earlier, will likely be very difficult to match using SE methods. However, because SE methods can be tailored to address specific issues—for example, PM6-ORG was designed to model details of specific biochemical interest—a combination of experimental and computational chemistry could produce geometries that could potentially be more accurate than experiment alone.

The semiempirical procedure to accomplish this is straightforward: starting with the experimental geometry, a global geometry optimization would be performed in which a bias towards the experimental geometry would be applied; this retains the accuracy of the overall structure and improves the accuracy of the fine details, such as hydrogen atom positions and interatomic bond lengths. As an example, all S–H bond lengths in the PDB geometry for 7JUN are less than 1.0 Å, i.e., about 0.4 Å less than the expected value. When the geometry of 7JUN was optimized, these bond lengths increased to their expected values. This discrepancy in the PDB geometry was then detected and reported when the differences between the PDB and the optimized geometry were identified using the “compare” keyword option in MOPAC.

By performing this test, potential errors in experimentally determined PDB structures could be identified and the appropriate corrections made.

### Deriving reference data for ΔH_f_ from ab initio energies

Given that, although the WebBook is a good source of accurate thermochemical data, the fact that a small fraction of them is of questionable accuracy for use as reference data raises the question: is there an alternative? One option being explored [50–53] would be the use of ab initio results to generate data that would have sub-kcal mol^−1^ accuracy; adopting this strategy appears to be a promising line of inquiry. Only one possible issue might complicate this approach, and that would be confirming that the accuracy is good when applied to compounds of elements that have no gas-phase exemplars.

### Importance of semiempirical methods

Several examples have been presented here showing how SE methods can be useful in modeling chemical systems, detecting possible errors in reference data sets, in modeling systems of biological importance, simulating reactions, solids, etc., all of which could have been done using other, more accurate methods, but there has been little or no indication of that happening. It was only when SE methods were used and attention was focused on them that the errors in the NIST WebBook became obvious. Similarly, the ambiguous nature of the lipid SQU-701 in PDB entry 1C3W was revealed only when that system was modeled.

The common thread is the advantage of the high speed of SE calculations, which permits large systems and large surveys to be run in a practicable time using only standard desktop hardware. Each entry in the WebBook would take only a few seconds to run, and, because comparison of predicted quantities with reference data can be done automatically using PARAM, analysis to identify possible problems can be automated.

This common thread could also be used in modeling biochemical phenomena as a preparatory step before more accurate methods are used. For example, when working with large biomolecules, a SE global optimization with a bias could provide a better starting point than the experimental structure. Then, when modeling complicated systems such as enzyme-catalyzed reaction mechanisms, an initial exploration of the various steps, ionization states, transition states, and other phenomena would provide valuable information mapping out what could be lying ahead. Any unexpected issues could be explored at little cost. All this could be done prior to any more time-consuming modeling being attempted.

## Conclusion

A robust set of software tools has been developed for optimizing values of parameters in semiempirical methods and for determining the accuracy of potential new methods by comparison with existing methods. Suggestions have been made regarding how their accuracy could be improved, but, even in their present form, they have proven effective in modeling some biochemical processes, as has been demonstrated by validation of the methods, investigating problems, detecting errors, and predicting the ability of ligands to inhibit enzyme activity.

Hopefully, the current usefulness of semiempirical methods will stimulate the development of even better ones. To facilitate this, all the software tools used in method development, both as source code and as executables, together with instructions and examples of their use, are provided in the supporting material.

## Supplementary Information

Below is the link to the electronic supplementary material.

ESM1 (ZIP 34.0 MB)

## Data Availability

A zip file in Supplementary Material contains all the material for setting up the parameterization of a newsemiempirical method, together with a worked example, and the tools used for comparing its accuracy with that of other methods.
