# Supplementary material for: Computational chemistry methods based on MNDO and tools for improving their accuracy
Source: J Mol Model. 2026 Mar 13;32(4):98. doi: 10.1007/s00894-026-06653-3 (PMC12987868; doi:10.1007/s00894-026-06653-3)
Supplement: Supplementary file 1 — ESM1 (ZIP 34.0 MB) [file 894_2026_6653_MOESM1_ESM.zip › README.pdf]

On-line Documentation for PARAM

Recommended procedure for setting up a starting environment

- All method development work should be done on a Windows 10 or 11 operating system.
- Create a folder with an appropriate name, for example PARAM. This will be used to contain all the material used in optimizing parameter values.
- Unzip the contents of the ZIP file into this folder.
- Review the command files. Each command file illustrates a specific action.
- Run the command file "Construct all.cmd" This will set up a complete working environment for method development. Time required: ~15 hours.
- Before proceeding further, carefully examine the new files and verify that the contents make sense.

Parameter optimization

A single command, "Method-development - Improve PM6-ORG.cmd", will run the parameter optimization process. The example given uses 3306 reference data, and optimizes 318 parameters. Once started, the rate of convergence of the parameters can be monitored, and when appropriate, the job should be killed, otherwise it would run for many days, even weeks. As soon as the job is understood, this command should be modified to use the current project.

Caveats

- The main collection of reference data, i.e., the set "Data Normal", has not been curated, and should be regarded as being of limited reliability. Warnings such as "Deadly error detected ..." and "ALL CONVERGERS ..." when all of "Data Normal" is used can be ignored.
- Conversely, the reference data files in the data-sets used in method development, i.e., those mentioned in PM6-ORG.dat such as "core.txt", have been curated. This was a necessary step in order to ensure that the values of the parameters could be optimized correctly. Errors that were ignored when all of "Data Normal" was used should *not* be ignored when method development is being done.
- In method development, individual data that take a long time, 100 or more seconds, to calculate should be deleted unless they are deemed necessary for the new method.
