# Supplementary material for: Computational chemistry methods based on MNDO and tools for improving their accuracy
Source: J Mol Model. 2026 Mar 13;32(4):98. doi: 10.1007/s00894-026-06653-3 (PMC12987868; doi:10.1007/s00894-026-06653-3)
Supplement: Supplementary file 1 — ESM1 (ZIP 34.0 MB) [file 894_2026_6653_MOESM1_ESM.zip › Build PARAM PM6-ORG Method development and Surveys/Data Intermolecular interactions/Water dimers Intermolecular/water_dimer_02.pdf]

JOURNAL OF CHEMICAL PHYSICS VOLUME 116, NUMBER 2 8 JANUARY 2002

Anchoring the water dimer potential energy surface with explicitly correlated computations and focal point analyses

Gregory S. Tschumper[a)]
Department of Chemistry and Biochemistry, University of Mississippi, University, Mississippi 38677

Matthew L. Leininger
Sandia National Laboratories, MS 9217, Livermore, California 94551

Brian C. Hoffman
Department of Chemistry, The Johns Hopkins University, Baltimore, Maryland 21218

Edward F. Valeev
Center for Computational Molecular Science and Technology, School of Chemistry and Biochemistry, Georgia Institute of Technology, Atlanta, Georgia 30332

Henry F. Schaefer III[b)]
Center for Computational Quantum Chemistry, University of Georgia, Athens, Georgia 30602-2525

Martin Quack
Laboratorium für Physikalische Chemie, ETH Hönggerberg, HCI, CH-8093 Zürich, Switzerland

(Received 28 June 2001; accepted 14 August 2001)

Ten stationary points on the water dimer potential energy surface have been characterized with the coupled-cluster technique which includes all single and double excitations as well as a perturbative approximation of triple excitations [CCSD(T)]. Using a triple-ζ basis set with two sets of polarization functions augmented with higher angular momentum and diffuse functions [TZ2P(f,d)+dif], the fully optimized geometries and harmonic vibrational frequencies of these ten stationary points were determined at the CCSD(T) theoretical level. In agreement with other *ab initio* investigations, only one of these ten stationary points is a true minimum. Of the other nine structures, three are transition structures, and the remaining are higher order saddle points. These high-level *ab initio* results indicate that the lowest lying transition state involved in hydrogen interchange is chiral, of C_1 symmetry rather than C_s as suggested by recently developed 6D potential energy surfaces. The one- and n-particle limits of the electronic energies of these ten stationary points were probed by systematic variation of the atomic orbital basis sets and the treatment of electron correlation within the framework of the focal-point analysis of Allen and co-workers. The one-particle limit was approached via extrapolation of electronic energies computed with the augmented correlation consistent basis sets (aug-cc-pVXZ, $X=D-6$), and, independently, by estimating the basis set incompleteness effect with the explicitly-correlated second-order Møller-Plesset method (MP2-R12). Electron correlation was evaluated at levels as high as the Brueckner coupled cluster method with double excitations and perturbatively treated triple and quadruple excitations [BD(TQ)]. Core correlation and relativistic effects were also assessed. Consideration of the aforementioned electronic effects as well as basis set superposition error leads to an estimate of 21.0 kJ mol^{-1} for the electronic dissociation energy of $(H_2O)_2$. © *2002 American Institute of Physics.* [DOI: 10.1063/1.1408302]

I. INTRODUCTION

The relative simplicity of the water molecule belies the extraordinary complexity of this material in the bulk phase. The properties of ice, liquid water, and water vapor are truly remarkable and of the utmost importance in a host of chemical and biological processes.[1–3] "Ubiquitous" is the favorite adjective of many chemists when describing this substance. A quick search through the literature will dispel any doubts that such a characterization might be an exaggeration. So prevalent is water in our daily lives that many of its remarkable physical properties are often overlooked. The boiling point of water is nearly 200 K above that of nonhydrogen bonded species with similar molecular weights. Also anomalously large for such a small molecule is its heat capacity in the liquid phase which is approximately twice that of ethanol (with a molecular weight more than two and a half times larger than water). Even more intriguing is the maximum density of water near 4 °C, implying that between 0 and 4 °C liquid water contracts upon heating. A universal, unified equation of state[4] based not just on empirical observation written in compact form but rather on a fundamental theoretical understanding is clearly of interest.

[a)]Postdoctoral Fellow 2000, Laboratorium für Physikalische Chemie, ETH Zürich (Zentrum) CH-8092 Zürich, Switzerland.
[b)]Author to whom correspondence should be addressed.

Downloaded 16 Feb 2003 to 130.207.165.29. Redistribution subject to AIP license or copyright, see http://ojps.aip.org/jcpo/jcpcr.jsp

Intimately related to these properties of water are the intermolecular interactions collectively referred to as hydrogen bonds. The massive amount of information on hydrogen bonding in the literature attests not only to the pervasive nature of this phenomenon in nearly every field of chemistry, but also the effort put forth by the scientific community seeking a deeper understanding of it. A thorough literature review of the subject is far beyond the scope of this or any other paper. Fortunately, several recent books[5–10] and review articles[11–13] are devoted entirely to hydrogen bonding. There is, of course, a wealth of information available from more classic sources that should not be overlooked.[1,14,15]

The hydrogen bond has only grudgingly revealed its innermost secrets. Our current knowledge of hydrogen bonding, while enormous, is far from complete.[16,17] One of the most productive approaches to this problem has been a kind of reductionism. By progressing from the simplest systems to exhibit hydrogen bonding, namely dimers, toward more complex systems such as trimers, tetramers, etc., it is possible to develop an understanding of the interactions between molecules and eventually the properties of the bulk phase.[17,18] Clearly, a quantitative, complete-dimensional, analytical intermolecular potential energy (hyper)surface (IPES) on which the quantum dynamics of hydrogen bonds can be studied is highly desirable since it thoroughly describes the forces between the molecules involved in hydrogen bonding[17] (within the limits of the Born–Oppenheimer approximation). Indeed, by systematically deriving a many body expansion of the potential, one may hope to get convergence at a modest order, say 5-body or at most 10-body terms, to describe the condensed phases. The starting point of such an approach must be a very accurate description of the 2-body (i.e., pair) potential. In practice, however, this has only been realized for the simplest of hydrogen bonded homodimers, namely $(HF)_2$ with six degrees of freedom.[19–22] The wealth of knowledge reaped from these labors has had a significant impact on our understanding of the spectroscopy and dynamics of hydrogen bonding.[23–28]

Since the construction of the first *ab initio* $(H_2O)_2$ IPES of Matsuoka, Clementi, and Yoshimine,[29] electronic structure theory has proven to be an invaluable tool in the construction of these surfaces.[19–22,30,31] In principle, quantum chemical computations allow chemists to arbitrarily examine any region of the IPES. The entire surface can be mapped out point by point. Furthermore, *ab initio* quantum chemical techniques are ideally suited for the "reductionist" approach. The progression to larger, more complex oligomers is limited only by computer resources and the tenacity of the investigating scientists.

In retrospect it is not surprising that the two most widely studied dimers have been those of water and hydrogen fluoride. The relative simplicity of these two prototypes makes them ideal for theoretical studies. Since the first *ab initio* investigations[32–35] of $(H_2O)_2$ and $(HF)_2$, these two systems have been the subject of numerous theoretical studies. With only two heavy atoms, highly sophisticated treatments of electron correlation can be employed in conjunction with massive basis sets when studying these dimers within the supermolecule framework.[36–39] These two dimers are also readily interrogated via fundamentally different approaches such as intermolecular or symmetry adapted perturbation theories (IMPT and SAPT, respectively).[31,40] As recently pointed out in the *Encyclopedia of Computational Quantum Chemistry*,[3] such investigations of $(H_2O)_2$ have focused on the global minimum and essentially ignored the rest of the IPES:

"However, to understand the most recent high-resolution experiments on the water dimer, trimer, tetramer, pentamer, and hexamer requires detailed consideration of the rearrangement pathways, of which there have been relatively few theoretical studies."[3]

The most thorough *ab initio* study to date of these rearrangement pathways of $(H_2O)_2$ was performed in 1990 by Smith *et al.*[41] They employed second-order Møller–Plesset perturbation theory (MP2) to characterize ten stationary points on the water dimer IPES. Three transition states, denoted as structures #2, #4, and #9, were found which connect the eight equivalent global minima (obtained by interchanging the hydrogen atoms within the water dimer via rotations or tunneling). For select stationary points, refined electronic energies were computed via a fourth-order Møller–Plesset perturbation theory (MP4). Results from recently constructed 6D water dimer potentials[42,43] suggest that some of the structures and barrier heights associated with the transition states reported by Smith *et al.* may be incorrect. Clearly more accurate *ab initio* data are needed if an IPES for $(H_2O)_2$ is ever to enjoy the reliability of those for $(HF)_2$.[20–22]

The primary, but not sole, goal of this work is to "anchor" the $(H_2O)_2$ IPES by characterizing ten stationary points with a target accuracy which approaches the current *ab initio* limit for this weakly bound dimer (± 0.2 kJ mol^{-1}).[39] The prescription for obtaining this goal has already appeared for the minimum energy configuration of $(H_2O)_2$ (referred to as structure #1 in this exposition).[37,38] The procedure is, in essence, an independent application of the focal point analysis (FPA) technique developed by Allen and co-workers.[44–50] For brevity, we merely borrow their own description of the FPA approach:

"whose characteristics generally include: (a) use of a family of basis sets which systematically approaches completeness (e.g., the cc-pVXZ, aug-cc-pVXZ, and cc-pCVXZ sets); (b) application of low levels of theory with prodigious basis sets (typically RHF and MP2 computations with several hundred basis functions); (c) higher-order valence correlation treatments [CCSDT, CCSD(T), BD(TQ), MP4, and MP5] with the largest possible basis sets; (d) layout of a two-dimensional extrapolation grid based on an assumed additivity of correlation *increments* to the energy difference of concern; and (e) eschewal of empirical corrections."[49]

Readers should consult the original references for more complete details, additional examples, evaluations of the performance of various extrapolation schemes and comparisons to other model chemistries (e.g., G3, W2, etc.) capable of high accuracy (often referred to as "subchemical" accuracy).

The analyses of the water dimer global minimum presented in Refs. 37–39 suggest that estimates of core–core and core–valence interaction are required in a FPA to reach an accuracy of a few tenths of a kJ mol^{-1}. FPA techniques

Downloaded 16 Feb 2003 to 130.207.165.29. Redistribution subject to AIP license or copyright, see http://ojps.aip.org/jcpo/jcpcr.jsp

TABLE I. A description of the basis sets used in this study. The last column lists the total number of basis functions for $(H_2O)_2$.

Basis sets	O contraction	H contraction	Number of B.F.
TZ2P(f,d)+dif	$(11s7p2d1f/6s4p2d1f)$	$(6s2p1d/4s2p1d)$	130
aug-cc-pVDZ	$(10s5p2d/4s3p2d)$	$(5s2p/3s2p)$	82
aug-cc-pVTZ	$(12s6p3d2f/5s4p3d2f)$	$(6s3p2d/4s3p2d)$	184
aug-cc-pVQZ	$(14s7p4d3f2g/6s5p4d3f2g)$	$(8s4p3d2f/5s4p3d2f)$	344
aug-cc-pV5Z	$(15s8p5d4f3g2h/7s6p5d4f3g2h)$	$(9s5p4d3f2g/6s5p4d3f2g)$	574
aug-cc-pV6Z	$(17s11p6d5f4g3h2i/9s8p6d5f4g3h2i)$	$(11s6p5d4f3g2h/7s6p5d4f3g2h)$	866
aug′-cc-pVDZ	same as aug-cc-pVDZ	$(4s1p/2s1p)$	66
aug′-cc-pVTZ	same as aug-cc-pVTZ	$(5s2p1d/3s2p1d)$	148
aug′-cc-pVQZ	same as aug-cc-pVQZ	$(6s3p2d1f/4s3p2d1f)$	280
aug′-cc-pV5Z	same as aug-cc-pV5Z	$(8s4p3d2f1g/5s4p3d2f1g)$	474
aug′-cc-pV6Z	same as aug-cc-pV6Z	$(10s5p4d3f2g1h/6s5p4d3f2h1h)$	742
aug-cc-pCVDZ	$(11s6p2d/5s4p2d)$	same as aug-cc-pVDZ	90
aug-cc-pCVTZ	$(13s8p4d2f/7s6p4d2f)$	same as aug-cc-pVTZ	210
aug-cc-pCVQZ	$(16s10p6d4f2g/9s8p6d4f2g)$	same as aug-cc-pVQZ	402
aug-cc-pCV5Z	$(19s13p8d6f4g2h/11s10p8d6f4g2h)$	same as aug-cc-pV5Z	682
$K2$[a]	$(15s9p7d5f)$	$(9s7p5d)$	508

[a]Uncontracted basis set for MP2-R12.

have pushed the technical limits of *ab initio* quantum chemistry even further in pursuit of spectroscopic accuracy (i.e., 1 cm^{-1} or 0.01 kJ mol^{-1}). To approach such an ambitious goal, relativistic and core correlation effects as well as the Born–Oppenheimer (BO) diagonal correction play a crucial role, even for molecules with only light atoms (F or smaller). Fortunately, the target accuracy of the present study is an order of magnitude larger than spectroscopic accuracy. Consequently, non-BO effects are neglected.

II. THEORETICAL METHODS

The theoretical models employed in this study of the water dimer intermolecular potential energy surface (IPES) can be divided into several distinct sections. First, the geometries of the ten stationary points of interest have been optimized via analytic gradient techniques. Vibrational frequencies have also been computed in order to characterize each point as a minimum, transition state or higher-order saddle point. In the second portion of the investigation, these geometries remained fixed while both the basis set and theoretical treatment of electron correlation were varied in order to approach the *ab initio* one- and *n*-particle limits of the relative energies of the ten stationary points. Core correlation and relativistic effects have also been evaluated. All basis sets employed in this investigation used spherical harmonic Gaussian functions.

A. Geometry optimization and vibrational analysis (Ref. 51)

Each of the ten stationary points has been optimized using the coupled-cluster method which explicitly includes all single and double excitations[52–55] as well as a noniterative, perturbative approximation of connected triple excitations[56] [i.e., the popular CCSD(T) method]. For the molecular orbital techniques employed in this study, the wave function is expanded in a set of atomic Gaussian basis functions. The basis chosen for geometry optimizations and frequency calculations is the TZ2P(f,d)+dif basis. This basis set consists of Dunning's triple-ζ contraction[57] of Huzinaga's primitive Gaussian functions,[58] $(5s/3s)$ for H and $(10s6p/5s3p)$ for O, augmented with two sets of polarization functions with orbital exponents $\alpha_p(\mathrm{H})=1.5$ and 0.375, and $\alpha_d(\mathrm{O})=1.7$ and 0.425 as well as one set of *f*-like higher angular momentum functions[59] for each O atom, $\alpha_f(\mathrm{O})=1.40$, and one set of *d*-like higher angular momentum functions[59] for each H atom, $\alpha_d(\mathrm{H})=1.0$. To this was added one set of even-tempered diffuse functions[60] with orbital exponents $\alpha_s(\mathrm{H})=0.030\,16$, $\alpha_s(\mathrm{O})=0.089\,93$, and $\alpha_p(\mathrm{O})=0.058\,40$. This and all other basis sets utilized in this investigation are summarized in Table I.

For each stationary point, full geometry optimizations were performed in the point group of each structure as determined in Ref. 41 and as shown in Fig. 1. Residual Cartesian gradients were less than 3.5×10^{-8} E_h/bohr. To characterize the nature of each stationary point, harmonic vibrational frequencies and their corresponding infrared (IR) intensities (double harmonic approximation) were determined via finite differences of analytic gradients.[61]

All of the coupled-cluster computations performed during the optimization of the geometries and the determination of the harmonic vibrational frequencies were carried out with the ACES II *ab initio* program package.[62] To take advantage of the analytic gradients for the CCSD(T) method,[63] all electrons were correlated and no orbitals were detected. From work on the HF dimer at the CCSD(T) level with similar basis sets, core correlation is expected to have a negligible effect on the geometry, vibrational frequencies and relative energies of these stationary points.[64]

B. Focal point analysis

In the second portion of the study, the relative energetics of these ten stationary points on the IPES at the *ab initio* limit were determined via the focal point analysis techniques mentioned earlier. All of the energy point computations were performed with the optimized structures obtained by the methods described in Sec. II A.

Two families of basis sets, denoted aug-cc-pV*XZ* and aug′-cc-pV*XZ* ($X=D$, T, Q, 5, 6), were employed to pursue

Downloaded 16 Feb 2003 to 130.207.165.29. Redistribution subject to AIP license or copyright, see http://ojps.aip.org/jcpo/jcpcr.jsp

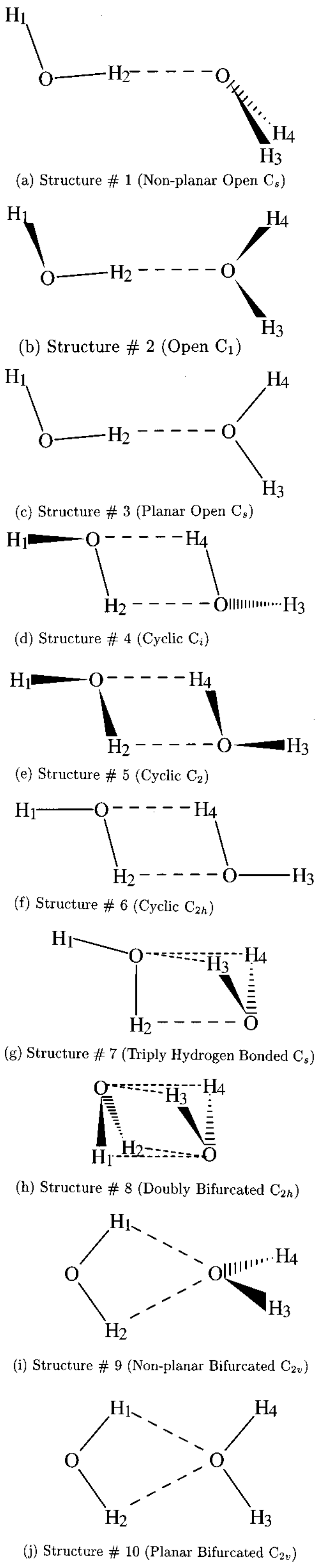

FIG. 1. The structures of all ten stationary points studied on the $(H_2O)_2$ intermolecular potential energy surface. O_1 is implicitly associated with the monomer containing H_1 and H_2 while O_2 is similarly associated with H_3 and H_4.

the one-particle basis set limits. The aug-cc-pXVZ basis sets[65] were formed by adding to Dunning's correlation consistent double, triple, quadruple, pentuple and sextuple polarized valence basis sets (cc-pVXZ) (Ref. 66) one diffuse function for each value of the angular momentum, l, for all atoms. The aug′-cc-pVXZ family, however, only adds the set of diffuse functions to the oxygen atoms (i.e., aug-cc-pVXZ for O atoms and cc-pVXZ for H atoms).

For each optimized structure, the electronic correlation energy was determined with three different post-Hartree–Fock procedures. The first is second order Møller–Plesset perturbation theory (MP2).[67,68] Also computed is the CCSD(T) energy which was described earlier. The final post-Hartree–Fock procedure used in the focal point analysis is the Brueckner doubles[69] technique which includes a perturbative treatment of both triple and quadruple excitations, denoted BD(TQ).[70] These energy point computations were carried out with the MPQC (Refs. 71–73) and GAUSSIAN 94 (Ref. 74) quantum chemical software packages. Unless noted otherwise, the O 1s-like core orbitals were excluded from all correlation procedures (i.e., frozen). All MP2 and CCSD(T) energies were converged to $1\times 10^{-10}\,E_h$ while a slightly less stringent convergence criterion of $10^{-7}\,E_h$ was used for the more computationally expensive BD(TQ) computations.

The one- and n-particle limits of these stationary points were determined by systematically varying both the basis set and theoretical models while the geometries remained fixed. Such an approach allows one to assess the contributions of higher excitations on the relative energies of various structures,

$$\delta E_{\mathrm{MP2}} = \Delta E_{\mathrm{MP2}} - \Delta E_{\mathrm{SCF}}, \quad (1)$$

$$\delta E_{\mathrm{CCSD(T)}} = \Delta E_{\mathrm{CCSD(T)}} - \Delta E_{\mathrm{MP2}}, \quad (2)$$

$$\delta E_{\mathrm{BD(TQ)}} = \Delta E_{\mathrm{BD(TQ)}} - \Delta E_{\mathrm{CCSD(T)}}. \quad (3)$$

For convergent theories, the n-particle limit is typically reached quickly for systems with a simple electronic structure. That is, contributions from higher excitations will rapidly approach zero. Empirically established convergence properties of the SCF and MP2 energies provide the means to extrapolate to the complete one-particle basis set limit. The complete basis set (CBS) SCF limit was obtained by fitting the SCF total electronic energies for $X=D,T,Q$,5,6 to a three-parameter function,[75,76]

$$E = E_{\mathrm{SCF}}^{\mathrm{CBS}} + a\ \exp(-bX). \quad (4)$$

Various formulae have been proposed to describe the convergence behavior of the correlation energy. Here we use the simple two-parameter formula suggested by Helgaker *et al.*,[77]

$$E = E_{\mathrm{MP2}}^{\mathrm{CBS}} + \frac{b}{X^3}. \quad (5)$$

One MP2 CBS limit was determined by fitting the $X=T,Q$,5,6 data to the above expression. To obtain a crude estimate of the error associated with such an extrapolation, we also evaluated the MP2 CBS limit by fitting just the two most accurate data points ($X=5$,6) to Eq. (5) using

$$E_{\mathrm{MP2}}^{\mathrm{CBS}} = \frac{E_X X^3 - E_{X-1}(X-1)^3}{X^3-(X-1)^3}. \quad (6)$$

Downloaded 16 Feb 2003 to 130.207.165.29. Redistribution subject to AIP license or copyright, see http://ojps.aip.org/jcpo/jcpcr.jsp

The explicitly correlated MP2 method of Kutzelnigg and Klopper[78] (MP2-R12) provides an independent means for obtaining the MP2 one-particle limit. That is achieved by including explicit linear dependence on the interelectronic distances in the first order correction to the reference wave function. Here we used the MP2-R12 method in standard approximation A (Ref. 79) as implemented in the PSI3 program package.[80,81] The approximate resolution of the identity implicit in the MP2-R12/A method in its present formulation demands basis sets of near Hartree–Fock limit quality to be used in computations. To this end, we use a specialized, uncontracted basis set, denoted K2, derived from Dunning's cc-pV5Z set by Klopper.[82] It is technically $(15s9p7d5f)$ and $(9s7p5d)$ for oxygen and hydrogen atoms, respectively. Thus, the K2 MP2-R12/A correlation energy is adopted as an estimate for the one-particle limit of the MP2 correlation energy. A MP2 CBS limit is then straightforwardly evaluated as a sum of the K2 MP2-R12/A correlation energy and the SCF CBS limit. In the remainder of this manuscript MP2-R12/A will simply be referred to as MP2-R12.

To assess the effects of core–core and core–valence correlation, the augmented polarized core/valence family of basis sets (aug-cc-pCVXZ, where $X=D,T,Q,5$) was employed.[83] The difference between the all electron and frozen core (*vide supra*) MP2 energies provides a good estimate of core correlation effects for a given basis set,

$$\delta E_{+\text{Core}} = \Delta E_{\text{MP2(all)}} - \Delta E_{\text{MP2(f.c.)}}. \tag{7}$$

For each structure, the standard first-order relativistic corrections to the energy (mass-velocity and Darwin terms) were obtained at the cc-pCVTZ CCSD(T) level of theory with the ACES II program package.

Although the aug-cc-V6Z basis sets can be considered complete under most circumstances, the target accuracy of this study mandates the consideration of basis set superposition error (BSSE).[84] The dissociation energy (D_e) of the global minimum structure of $(H_2O)_2$ into two H_2O monomers is computed with and without a counterpoise (CP) correction.[85,86] The notation $E_G^B(F)$ is introduced to denote the basis set (B) and geometry (G) used to compute the energy of fragment F. The uncorrected D_e is merely the energy of two water monomers ($A=B$) minus the energy of the water dimer (AB),

$$D_e = 2\times E_A^A(A) - E_{AB}^{AB}(AB). \tag{8}$$

The correction for BSSE allows the monomers to utilize the basis functions available in the dimer and compensates for relaxation effects,

$$\begin{aligned} D_e^{\text{CP}} &= D_e + \Delta E^{\text{CP}} \\ &= E_A^A(A) + E_B^B(B) - E_{AB}^{AB}(AB) \\ &\quad + [E_{AB}^{AB}(A) + E_{AB}^{AB}(B) - E_{AB}^{A}(A) - E_{AB}^{B}(B)]. \end{aligned} \tag{9}$$

The major drawback of this procedure is the additional computational effort required. The monomer computations in the dimer basis are nearly as time consuming as those for the dimer. Since the energy of two monomers is compared only to the global minimum, this correction is applied only for that structure. All other energy differences are not CP corrected.

III. RESULTS AND DISCUSSION

A. Structural and vibrational analyses

The structures of the ten stationary points are displayed in Fig. 1. The optimized geometrical parameters[51] determined at the CCSD(T) level of theory with the TZ2P(f,d) +dif basis are given in Tables II and III for the intra- and intermonomer variables, respectively. Some redundant parameters have been included to facilitate reproduction of the structures. The corresponding Cartesian coordinates are reported to eight decimal places in the supplementary material accompanying this paper.[87]

A quick glance at Tables II and III reveals no gross qualitative differences between the ten structures reported by Smith *et al.*[41] and those obtained in this study with a much larger basis set and more extensive treatment of electron correlation. In addition, the Hessian index of each stationary point is also unchanged from the earlier study. Structure #1 remains the only minimum while structures #2, #4, and #9 are transition states. The other stationary points are higher-order saddle points with two or three imaginary vibrational frequencies. These are very important results in light of the recently developed SAPT-5s and SAPT-5st 6D potentials.[43] Transition state #2 apparently does not exist on either IPES. Instead, structure #3 of C_s symmetry was found to be the lowest transition state involved in the rearrangement between the 8 equivalent minima of structure #1. The CCSD(T)/TZ2P(f,d)+dif harmonic vibrational spectra[51] (including infrared intensities) provided in the supplementary material accompanying this paper[87] indicate that structure #3 is a second-order saddle point.

A more detailed inspection of Table III uncovers some substantial quantitative changes in the intermonomer geometrical parameters obtained in this study for structures with bifurcated hydrogen bonds (#7–#10) relative to those reported in Ref. 41. In the following discussion, all comparisons refer to changes in the MP2 optimized structures reported by Smith and co-workers[41] [obtained with 6-31+G(d,p) or 6-311+G(d,p) basis sets] after reoptimization at the CCSD(T) level with the TZ2P(f,d)+dif basis set. For the simple bifurcated structures (#9 and #10), the use of a larger basis set and inclusion of connected triple excitations shortens the hydrogen bonds by 0.05 Å. This effect is slightly smaller for the doubly bifurcated stationary points (#8). For structure #7, the bifurcated hydrogen bond length actually decreases by approximately 0.05 Å but is accompanied by a dramatic lengthening of the regular hydrogen bond ($r_{H_2O_2}$) by almost 0.4 Å. Since the OO distance of structure #7 increases by only 0.07 Å, the changes in hydrogen bond lengths can be primarily attributed to changes in the intermonomer bond angles. In the present study, H_3 and H_4 are inclined more directly toward O_1 as indicated by $\theta_{H_3O_2O_1}$ and $\theta_{H_4O_2O_1}$. In a similar vein but opposite direction, H_2 is angled further away from O_2 ($\theta_{O_1H_2O_2}$). As will be evident

Downloaded 16 Feb 2003 to 130.207.165.29. Redistribution subject to AIP license or copyright, see http://ojps.aip.org/jcpo/jcpcr.jsp

TABLE II. Optimized intramonomer geometrical parameters of H_2O and $(H_2O)_2$ obtained at the CCSD(T)/TZ2P(f,d)+dif level. Bond lengths (r_{XY}) are in Å and angles (θ_{XYZ}) are in deg. The values in parentheses correspond to the MP2 optimized structures reported in an earlier study.[a] The atom numbers correspond to those depicted in Fig. 1.

	Structure	$r_{O_1H_1}$	$r_{O_1H_2}$	$r_{O_2H_3}$	$r_{O_2H_4}$	$\theta_{H_1O_1H_2}$	$\theta_{H_3O_1H_4}$
H_2O	Monomer[b]	0.9589	0.9589	⋯	⋯	104.16	⋯
		(0.963)	(0.963)	⋯	⋯	(105.5)	⋯
#1	Nonplanar open C_s	0.9581	0.9653	0.9597	0.9597	104.45	104.58
		(0.958)	(0.965)	(0.960)	(0.960)	(103.6)	(104.1)
#2	Open C_1	0.9580	0.9645	0.9595	0.9589	104.47	104.84
		(0.958)	(0.964)	(0.960)	(0.959)	(103.7)	(104.4)
#3	Planar open C_s	0.9579	0.9640	0.9590	0.9585	104.48	105.04
		(0.962)	(0.968)	(0.963)	(0.963)	(105.7)	(106.2)
#4	Cyclic C_i	0.9585	0.9616	0.9586	0.9616	104.84	104.84
		(0.959)	(0.962)	(0.959)	(0.962)	(104.5)	(104.5)
#5	Cyclic C_2	0.9583	0.9614	0.9583	0.9614	104.95	104.95
		(0.962)	(0.966)	(0.962)	(0.966)	(106.4)	(106.4)
#6	Cyclic C_{2h}	0.9580	0.9611	0.9580	0.9611	105.14	105.14
		(0.962)	(0.965)	(0.962)	(0.965)	(106.5)	(106.5)
#7	Triply hydrogen bonded C_s	0.9591	0.9598	0.9598	0.9598	104.61	102.00
		(0.963)	(0.964)	(0.964)	(0.964)	(106.2)	(103.1)
#8	Doubly bifurcated C_{2h}	0.9594	0.9594	0.9594	0.9594	103.15	103.15
		(0.963)	(0.963)	(0.963)	(0.963)	(104.1)	(104.1)
#9	Nonplanar bifurcated C_{2v}	0.9596	0.9596	0.9593	0.9593	101.56	104.42
		(0.960)	(0.960)	(0.960)	(0.960)	(100.5)	(103.9)
#10	Planar bifurcated C_{2v}	0.9591	0.9591	0.9592	0.9592	101.91	104.09
		(0.963)	(0.963)	(0.963)	(0.963)	(102.2)	(105.2)

[a]From Ref. 41: 6-311+G(d,p) for #1, #2, #4, #9; 6-31+G(d,p) for all others.
[b]MP2/6-311+G(d,p) r_{OH}=0.959 Å and θ_{HOH}=103.5°.

from the rest of this section, these rather significant geometrical deviations have little affect on the relative energies of the species due to the very flat nature of the potential energy surface along these large amplitude, intermonomer coordinates. Neither the curvature nor the energy changes very rapidly as a function of these geometrical parameters.

To estimate the overall quality of the CCSD(T)/TZ2P(f,d)+dif optimized structures, we compare the geometrical parameters of the global minimum obtained in this investigation to other high level studies recently reported in the literature. Klopper *et al.* provide a more thorough survey of the literature.[39] Here we merely tabulate the

TABLE III. Optimized intermonomer geometrical parameters of $(H_2O)_2$ obtained at the CCSD(T)/TZ2P(f,d)+dif level. Bond lengths (r_{XY}) are in Å and angles (θ_{XYZ} and τ_{WXYZ}) are in deg. The values in parentheses correspond to the MP2 optimized structures reported in an earlier study.[a] The atom numbers correspond to those depicted in Fig. 1.

	Structure	$r_{H_2O_2}$	$\theta_{O_1H_2O_2}$	$\theta_{H_3O_2O_1}$	$\theta_{H_4O_2O_1}$	$\tau_{O_2H_2O_1H_1}$	$\tau_{H_3O_2O_1H_2}$	$\tau_{H_4O_2O_1H_2}$
#1	Nonplanar open C_s	1.9485	172.92	110.50	110.50	180.00	122.37	−122.37
		(1.944)	(176.7)	(116.2)	(116.2)	(180.0)	(118.5)	(−118.5)
#2	Open C_1	1.9724	168.97	107.33	135.00	144.61	159.63	25.88
		(1.970)	(170.6)	(110.2)	(134.7)	(137.4)	(172.3)	(34.9)
#3	Planar open C_s	1.9813	167.59	109.96	145.00	180.00	180.00	0.00
		(1.970)	(169.1)	(111.7)	(142.1)	(180.0)	(180.0)	(0.0)
#4	Cyclic C_i	2.2796	114.84	132.32	47.15	−134.78	111.86	180.00
		(2.278)	(112.3)	(138.0)	(49.1)	(−142.4)	(118.0)	(180.0)
#5	Cyclic C_2	2.2810	112.52	145.10	48.95	−153.28	−118.27	−167.68
		(2.273)	(109.6)	(152.6)	(51.1)	(−162.2)	(−132.5)	(−172.2)
#6	Cyclic C_{2h}	2.2756	110.27	155.80	50.66	180.00	180.00	180.00
		(2.276)	(108.5)	(158.5)	(52.0)	(180.0)	(180.0)	(180.0)
#7	Triply hydrogen bonded C_s	2.9997	77.32	55.16	55.16	180.00	−108.75	108.75
		(2.621)	(95.1)	(62.4)	(62.4)	(180.0)	(−117.8)	(117.8)
#8	Doubly bifurcated C_{2h}	3.1140	92.24	70.84	70.84	−64.02	180.00	67.93
		(3.144)	(90.9)	(72.2)	(72.2)	(−65.6)	(180.0)	(68.1)
#9	Nonplanar bifurcated C_{2v}	2.5154	112.03	127.79	127.79	0.00	−90.00	90.00
		(2.462)	(112.3)	(128.0)	(128.0)	(0.0)	(−90.0)	(90.0)
#10	Planar bifurcated C_{2v}	2.6830	112.92	127.95	127.95	0.00	0.00	180.00
		(2.630)	(112.3)	(127.4)	(127.4)	(0.0)	(0.0)	(180.0)

[a]From Ref. 41: 6-311+G(d,p) for #1, #2, #4, #9; 6-31+G(d,p) for all others.

Downloaded 16 Feb 2003 to 130.207.165.29. Redistribution subject to AIP license or copyright, see http://ojps.aip.org/jcpo/jcpcr.jsp

TABLE IV. A comparison of key geometrical parameters of the $(H_2O)_2$ global minimum (structure #1) obtained at various theoretical levels. Bond lengths (r_{XY}) are in Å and angles are in deg (θ_{XYZ}). $\theta_{O_1O_2\text{Bisec}}$ denotes the angle between the OO axis and the vector originating at O_2 and bisecting $\theta_{H_3O_2H_4}$. Parameters in square brackets were not optimized. The atom numbers correspond to those depicted in Fig. 1.

Method	Basis	$r_{O_1O_2}$	$r_{H_2O_1}$	$\theta_{O_2O_1H_2}$	$\theta_{O_1O_2\text{Bisec}}$
		ab initio			
CCSD(T)	TZ2P(f,d)+dif	2.9089	0.9653	4.74	124.92
MP2[a]	aug-cc-pVQZ	2.903	0.966	5.6	124.8
MP2[a,b]	aug-cc-pVQZ	2.917	0.966	5.1	124.7
CCSD(T)[c]	aug-cc-pVTZ	2.8954	[0.9572]	4.76	122.51
SAPT[d]		2.95	[0.9716]	4.6	124
Extrapolated[e]	∞	2.912	0.9639	5.5	124.4
		IPES			
VRT(ASP-W)[f]	⋯	2.924	[0.9572]	−2.3[g]	131.5
SAPT-5s[h]	⋯	2.955	[0.9716]	6.3	127
SAPT-pp[h]	⋯	2.945	[0.9716]	1.0	149

[a]Reference 88.
[b]Geometry optimization with CP corrected gradients.
[c]Reference 89: Constrained optimization using r_e for the monomers ($r_{OH}=0.9572$ Å and $\theta_{HOH}=104.52°$).
[d]Reference 43: Constrained optimization using r_0 for the monomers ($r_{OH}=0.971\,625\,7$ Å and $\theta_{HOH}=104.69°$.
[e]Reference 39.
[f]Reference 42: Equilibrium structure on the 6D VRT(ASP-W) IPES of $(D_2O)_2$.
[g]H_1 and H_2 are on opposite sides of OO axis rather than on the same side.
[h]Reference 43: Equilibrium structure on the 6D SAPT potentials.

most reliable and most common *ab initio* parameters as well as some very recent results from two site–site potentials fit to more than 2500 interaction energies computed with symmetry-adapted perturbation theory.[43] The intermonomer geometrical parameters most often used for structure #1 are $r_{O_1O_2}$, $\theta_{O_2O_1H_2}$, and $\theta_{O_1O_2\text{Bisec}}$ (which denotes the angle between OO axis and a vector that originates at O_2 and bisects $\theta_{H_3O_2H_4}$). As one can see from Table IV, the results obtained in this work agree very well with the counterpoise (CP) corrected MP2/aug-cc-pVQZ structure obtained by Hobza *et al.*[88] as well as the extrapolated structure in Ref. 39. This agreement clearly represents a fortuitous cancellation of errors since the basis set superposition error for the TZ2P(f,d)+dif basis is not negligible for either $(H_2O)_2$ or $(HF)_2$.[37,64] Nevertheless, it supports the previous observation[64] that the relatively modest TZ2P(f,d)+dif basis set can yield equilibrium structures for simple hydrogen bonded systems that are comparable to those obtained with the much larger cc-pVQZ and aug-cc-pVQZ type basis sets (including counterpoise correction). Thus, it is recommended that future investigations of the water dimer global minimum use the structure given here[51] or those given in Refs. 39 and 88 rather than the constrained CCSD(T)/aug-cc-pVTZ optimized geometry[89] that abounds in the literature.

B. Energy analysis

Table V shows the effects of electron correlation and basis set size on the energy of the minima and transition states relative to the global minimum (structure #1). The complete list of FPA tables for all stationary points is pro-

TABLE V. Effects of basis set size and electron correlation on the electronic energies (in kJ mol^{-1}) of minima and transition states on the water dimer PES relative to the global minimum (structure #1, nonplanar open C_s). ΔE_{SCF} is the energy difference at the SCF level while δE_{MP2}, $\delta E_{\text{CCSD(T)}}$, and $\delta E_{\text{BD(TQ)}}$ are the increments from MP2, CCSD(T), and BD(TQ) methods, respectively. ΔE_{corr} (in bold) is the correlated energy difference which includes the MP2, CCSD(T), and BD(TQ) contributions. Values in square brackets are assumed.

Basis	ΔE_{SCF}	δE_{MP2}	$\delta E_{\text{CCSD(T)}}$	$\delta E_{\text{BD(TQ)}}$	ΔE_{corr}
	Structure #2 (open C_1)				
TZ2P(f,d)+dif	1.60	+0.75	+0.05	⋯	**2.40**
aug-cc-pVDZ	1.69	+0.61	+0.07	−0.01	**2.36**
aug-cc-pVTZ	1.51	+0.69	+0.09	[−0.01]	**2.29**
aug-cc-pVQZ	1.47	+0.69	[+0.09]	[−0.01]	**2.25**
aug-cc-pV5Z	1.46	+0.67	[+0.09]	[−0.01]	**2.21**
aug-cc-pV6Z	1.46	+0.65	[+0.09]	[−0.01]	**2.19**
CBS[a,b]	1.46	+0.65	[+0.09]	[−0.01]	**2.19**
CBS[c]		+0.63			**2.17**
CBS[d]		+0.67			**2.21**
**ΔE$_{avg}$**[e]					**2.19±0.05**
aug′-cc-pVDZ	1.92	+0.60	+0.03	−0.01	**2.55**
aug′-cc-pVTZ	1.51	+0.71	+0.08	[−0.01]	**2.29**
aug′-cc-pVQZ	1.47	+0.67	+0.07	[−0.01]	**2.20**
aug′-cc-pV5Z	1.46	+0.65	[+0.07]	[−0.01]	**2.17**
aug′-cc-pV6Z	1.46	+0.64	[+0.07]	[−0.01]	**2.16**
CBS[a,b]	1.47	+0.62	[+0.07]	[−0.01]	**2.14**
CBS[c]		+0.61			**2.14**
CBS[d]		+0.67			**2.20**
**ΔE$_{avg}$**[e]					**2.16±0.06**
	Structure #4 (cyclic C_i)				
TZ2P(f,d)+dif	3.25	+0.46	−0.13	⋯	**3.58**
aug-cc-pVDZ	3.04	+0.69	−0.01	−0.01	**3.71**
aug-cc-pVTZ	3.16	+0.32	−0.10	[−0.01]	**3.36**
aug-cc-pVQZ	3.19	+0.10	[−0.10]	[−0.01]	**3.17**
aug-cc-pV5Z	3.17	+0.06	[−0.10]	[−0.01]	**3.11**
aug-cc-pV6Z	3.16	−0.02	[−0.10]	[−0.01]	**3.02**
CBS[a,b]	3.17	−0.04	[−0.10]	[−0.01]	**3.01**
CBS[c]		−0.15			**2.89**
CBS[d]		−0.17			**2.88**
**ΔE$_{avg}$**[e]					**2.93±0.16**
aug′-cc-pVDZ	3.38	+0.54	−0.01	−0.02	**3.89**
aug′-cc-pVTZ	3.04	+0.32	−0.11	[−0.02]	**3.24**
aug′-cc-pVQZ	3.18	+0.03	−0.13	[−0.02]	**3.06**
aug′-cc-pV5Z	3.18	−0.03	[−0.13]	[−0.02]	**2.99**
aug′-cc-pV6Z	3.16	−0.08	[−0.13]	[−0.02]	**2.94**
CBS[a,b]	3.21	−0.14	[−0.13]	[−0.02]	**2.92**
CBS[c]		−0.20			**2.86**
CBS[d]		−0.17			**2.89**
**ΔE$_{avg}$**[e]					**2.89±0.11**
	Structure #9 (nonplanar bifurcated C_{2v})				
TZ2P(f,d)+dif	5.19	+2.77	−0.31	⋯	**7.66**
aug-cc-pVDZ	5.16	+3.05	−0.28	−0.08	**7.85**
aug-cc-pVTZ	5.28	+3.09	−0.31	[−0.08]	**7.96**
aug-cc-pVQZ	5.29	+2.83	[−0.31]	[−0.08]	**7.72**
aug-cc-pV5Z	5.24	+2.79	[−0.31]	[−0.08]	**7.63**
aug-cc-pV6Z	5.23	+2.70	[−0.31]	[−0.08]	**7.53**
CBS[a,b]	5.23	+2.67	[−0.31]	[−0.08]	**7.50**
CBS[c]		+2.57			**7.41**
CBS[d]		+2.51			**7.35**
**ΔE$_{avg}$**[e]					**7.42±0.18**
aug′-cc-pVDZ	4.75	+2.26	−0.27	−0.04	**6.70**
aug′-cc-pVTZ	5.08	+2.82	−0.33	[−0.04]	**7.52**
aug′-cc-pVQZ	5.25	+2.68	−0.37	[−0.04]	**7.52**
aug′-cc-pV5Z	5.24	+2.64	[−0.37]	[−0.04]	**7.46**
aug′-cc-pV6Z	5.23	+2.62	[−0.37]	[−0.04]	**7.44**
CBS[a,b]	5.26	+2.61	[−0.37]	[−0.04]	**7.46**
CBS[c]		+2.56			**7.41**

Downloaded 16 Feb 2003 to 130.207.165.29. Redistribution subject to AIP license or copyright, see http://ojps.aip.org/jcpo/jcpcr.jsp

TABLE V. *(Continued.)*

Basis	ΔE_{SCF}	δE_{MP2}	$\delta E_{CCSD(T)}$	$\delta E_{BD(TQ)}$	ΔE_{corr}
CBS[d]		+2.51			**7.36**
**ΔE_{avg}**[e]					**7.41±0.14**
		2 H_2O (D_e^{CP})			
TZ2P(f,d)+dif	14.67	+4.58	−0.10	⋯	**19.15**
aug-cc-pVDZ	14.49	+4.01	−0.14	−0.08	**18.27**
aug-cc-pVTZ	14.38	+5.32	+0.16	[−0.08]	**19.78**
aug-cc-pVQZ	14.49	+5.84	[+0.16]	[−0.08]	**20.40**
aug-cc-pV5Z	14.48	+6.03	[+0.16]	[−0.08]	**20.59**
aug-cc-pV6Z	14.48	+6.13	[+0.16]	[−0.08]	**20.68**
CBS[a,b]	14.50	+6.24	[+0.16]	[−0.08]	**20.82**
CBS[c]		+6.24			**20.82**
CBS[d]		+6.29			**20.87**
**ΔE_{avg}**[e]					**20.84±0.32**
aug′-cc-pVDZ	14.79	+3.93	−0.35	−0.09	**18.28**
aug′-cc-pVTZ	14.37	+5.20	+0.06	[−0.09]	**19.54**
aug′-cc-pVQZ	14.45	+5.75	+0.17	[−0.09]	**20.28**
aug′-cc-pV5Z	14.48	+5.99	[+0.17]	[−0.09]	**20.55**
aug′-cc-pV6Z	14.48	+6.10	[+0.17]	[−0.09]	**20.66**
CBS[a,b]	14.51	+6.20	[+0.17]	[−0.09]	**20.80**
CBS[c]		+6.22			**20.82**
CBS[d]		+6.29			**20.88**
**ΔE_{avg}**[e]					**20.83±0.34**
		2 H_2O (D_e)			
TZ2P(f,d)+dif	15.08	+6.57	−0.06	⋯	**21.58**
aug-cc-pVDZ	15.51	+6.52	+0.16	−0.17	**22.02**
aug-cc-pVTZ	14.70	+6.96	+0.16	[−0.17]	**21.65**
aug-cc-pVQZ	14.61	+6.69	[+0.16]	[−0.17]	**21.29**
aug-cc-pV5Z	14.50	+6.56	[+0.16]	[−0.17]	**21.05**
aug-cc-pV6Z	14.48	+6.46	[+0.16]	[−0.17]	**20.93**
CBS[a,b]	14.50	+6.39	[+0.16]	[−0.17]	**20.88**
CBS[c]		+6.28			**20.77**
CBS[d]		+6.28			**20.77**
**ΔE_{avg}**[e]					**20.81±0.24**
aug′-cc-pVDZ	15.91	+5.75	−0.11	−0.17	**21.38**
aug′-cc-pVTZ	14.62	+6.44	+0.07	[−0.17]	**20.96**
aug′-cc-pVQZ	14.56	+6.33	+0.06	[−0.17]	**20.79**
aug′-cc-pV5Z	14.50	+6.30	[+0.06]	[−0.17]	**20.70**
aug′-cc-pV6Z	14.48	+6.30	[+0.06]	[−0.17]	**20.68**
CBS[a,b]	14.53	+6.22	[+0.06]	[−0.17]	**20.64**
CBS[c]		+6.22			**20.65**
CBS[d]		+6.28			**20.70**
**ΔE_{avg}**[e]					**20.66±0.21**

[a]ΔE_{SCF} complete basis set limit calculated from E_{SCF}^{CBS} extrapolations of Eq. (4).
[b]δE_{MP2} complete basis set limit calculated from E_{MP2}^{CBS} extrapolations of Eq. (5).
[c]δE_{MP2} complete basis set limit calculated from E_{MP2}^{CBS} extrapolations of Eq. (6).
[d]δE_{MP2} complete basis set limit calculated from MP2-R12 energies.
[e]Average of ΔE_{corr} CBS limits (see text for details).

TABLE VI. Effect of core correlation on the electronic energies (in kJ mol^{-1}) of minima and transition states on the water dimer PES relative to the global minimum (structure #1, nonplanar open C_s). $\Delta E_{MP2(f.c.)}$ and $\Delta E_{MP2(all)}$ are the energy differences from the MP2 frozen core and all electron calculations. δE_{+Core} is the increment obtained by correlating the core electrons.

Basis	$\Delta E_{MP2(f.c.)}$	$\Delta E_{MP2(all)}$	δE_{+Core}
	Structure #2 (open C_1)		
aug-cc-pCVDZ	2.30	2.32	+0.02
aug-cc-pCVTZ	2.17	2.17	+0.01
aug-cc-pCVQZ	2.13	2.13	+0.00
aug-cc-pCV5Z	2.11	2.11	−0.00
	Structure #4 (cyclic C_i)		
aug-cc-pCVDZ	3.73	3.86	+0.13
aug-cc-pCVTZ	3.38	3.42	+0.04
aug-cc-pCVQZ	3.24	3.26	+0.02
aug-cc-pCV5Z	3.17	3.20	+0.02
	Structure #9 (nonplanar bifurcated C_{2v})		
aug-cc-pCVDZ	8.23	8.39	+0.16
aug-cc-pCVTZ	8.17	8.27	+0.10
aug-cc-pCVQZ	8.03	8.10	+0.07
aug-cc-pCV5Z	7.96	8.03	+0.07
	2 H_2O (D_e^{CP})		
aug-cc-pCVDZ	18.39	18.43	+0.04
aug-cc-pCVTZ	19.76	19.90	+0.14
aug-cc-pCVQZ	20.45	20.61	+0.16
aug-cc-pCV5Z	20.65	20.82	+0.17
	2 H_2O (D_e)		
aug-cc-pCVDZ	22.07	22.38	+0.31
aug-cc-pCVTZ	21.38	21.58	+0.19
aug-cc-pCVQZ	21.16	21.31	+0.15
aug-cc-pCV5Z	20.97	21.12	+0.15

vided in the supplementary material.[87] All energy differences and energy increments are given in kJ mol^{-1}. Any inconsistencies in Tables V–VII are merely rounding errors since each entry has been computed directly from the electronic energies and rounded to two decimal places. The electronic energies are available in the supplementary material accompanying this paper.[87]

The complete basis set (CBS) SCF limit was obtained by fitting the SCF total electronic energies for $X=D,T,Q$,5,6 to Eq. (4). For all dimer stationary points as well as two water monomers, the SCF limit is essentially reached with both the aug-cc-pV6Z and aug′-cc-pV6Z basis sets. The CBS limits of the SCF energy difference (ΔE_{SCF}) differ from the aug-cc-pV6Z result by no more than 0.02 kJ mol^{-1} (for structure #6) and from the aug′-cc-pV6Z value by 0.06 kJ mol^{-1} (for structure #8).

Convergence to the MP2 CBS limit does not occur as quickly. The sextuple-ζ results differ from the three estimates of the MP2 CBS limit [MP2-R12 and Eqs. (5) and (6)] by as much as 0.2 kJ mol^{-1}. The massive aug-cc-pV6Z basis still suffers from a non-negligible basis set incompleteness error (not to be confused with basis set superposition error). The aug-cc-pV6Z and aug′-cc-pV6Z MP2 energies are always within 0.11 kJ mol^{-1} of the respective CBS values from Eq. (5). When one calculates the average of the three MP2 limits, none deviate from the mean by more than 0.10 kJ mol^{-1}.

Correlation plays a large role in the relative electronic energies of the stationary points. For some cases, the MP2 correction (δE_{MP2}) is larger than the SCF energy difference (ΔE_{SCF}). As expected, contributions from higher-order excitations diminish rapidly. For most structures, $\delta E_{CCSD(T)}$ is roughly an order of magnitude smaller than δE_{MP2} and the BD(TQ) correction [$\delta E_{BD(TQ)}$] smaller still. The contribution from connected quadruple excitations approaches or exceeds 0.1 kJ mol^{-1} only for bifurcated structures (#7–#10) and the water monomer. From the aug′-cc-pVTZ and aug′-cc-pVQZ data, it is clear that the contribution from triple excitations is

Downloaded 16 Feb 2003 to 130.207.165.29. Redistribution subject to AIP license or copyright, see http://ojps.aip.org/jcpo/jcpcr.jsp

TABLE VII. Summary of the ten water dimer stationary points studied in this investigation. Included are the number of imaginary vibrational frequencies (N_i), the dipole moment (μ_e) in Debye and the current best estimate of the electronic energy relative to the global minimum (ΔE_e) after including core correlation ($\delta E_{+\mathrm{Core}}$) and relativistic ($\delta E_{\mathrm{rel}}$) corrections. All energies are in kJ mol^{-1}.

	Structure	N_i[a]	μ_e[b]	ΔE_{avg}[c]	$\delta E_{+\mathrm{Core}}$[d]	δE_{rel}[e]	ΔE_e	MP4[f]	MP4[g]	VRT(ASP-W)[h]	SAPT-5s[i]	SAPT-pp[i]
#1	Nonplanar open C_s	0	2.6966	0.00	+0.00	+0.000	0.00±0.00	0.0	0.0	0.00	0.00	0.00
#2	Open C_1	1	3.7345	2.17	−0.00	+0.001	2.17±0.06	2.8	2.5	1.88	⋯	⋯
#3	Planar open C_s	2	3.4377	2.39	−0.02	+0.002	2.37±0.07	3.1	⋯	⋯	1.87	2.66
#4	Cyclic C_i	1	0.0000	2.91	+0.02	−0.001	2.93±0.18	4.2	3.6	2.48	2.21	2.97
#5	Cyclic C_2	2	1.7183	3.97	+0.02	−0.001	3.98±0.18	5.5	⋯	⋯	⋯	⋯
#6	Cyclic C_{2h}	3	0.0000	4.16	−0.00	+0.001	4.16±0.21	5.7	⋯	⋯	⋯	⋯
#7	Triply hydrogen bonded C_s	2	3.4057	7.52	+0.07	−0.003	7.59±0.21	7.7	⋯	⋯	⋯	⋯
#8	Doubly bifurcated C_{2h}	3	0.0000	14.82	+0.13	−0.006	14.94±0.25	15.7	⋯	⋯	⋯	⋯
#9	Nonplanar bifurcated C_{2v}	1	4.1535	7.41	+0.07	−0.003	7.48±0.19	7.5	7.9	4.71	7.60	8.19
#10	Planar bifurcated C_{2v}	2	4.1121	11.26	+0.08	−0.003	11.34±0.27	11.9	⋯	⋯	⋯	⋯
2 H_2O	Monomers (CP corrected)	⋯	⋯	20.83	+0.17	−0.010	20.99±0.34	⋯	18.7	⋯	⋯	⋯
2 H_2O	Monomers	0	1.9250	20.73	+0.15	−0.008	20.88±0.32	22.4	22.6	20.5	20.34	21.04

[a]Number of imaginary frequencies at the CCSD(T)/TZ2P(f,d)+dif level.
[b]Dipole moment with respect to the center-of-mass at the CCSD(T)/TZ2P(f,d)+dif level.
[c]ΔE_{avg} using both the aug-cc-pVXZ and aug′-cc-pVXZ series from Table V.
[d]Core correlation correction at the MP2/aug-cc-pCV5Z level from Table VI.
[e]Relativistic correction at the CCSD(T)/cc-pCVTZ level.
[f]MP4/6-31+G($2d$,$2p$) energies for MP2/6-31+G(d,p) optimized structures from Ref. 41.
[g]MP4/6-311+G($2df$,$2p$) energies for MP2/6-311+G(d,p) optimized structures from Ref. 41.
[h]Reference 42: 6D VRT(ASP-W) IPES of $(D_2O)_2$.
[i]Reference 43: 6D SAPT potentials.

close to convergence with respect to the cardinal number of the basis sets. The deviation in $\delta E_{\mathrm{CCSD(T)}}$ between these two basis sets is less than 0.05 kJ mol^{-1} for all relative energies except the CP corrected dissociation energy and is generally much smaller than the deviation between the aug-cc-pVDZ and aug-cc-pVTZ basis sets. It is also worth mentioning that the correlation contributions do not necessarily converge uniformly. Careful analysis of Table V (and the corresponding supplementary material[87]) reveals nonuniform convergence of the MP2 increments. For example, the difference between the aug-cc-pVQZ and aug-cc-pV5Z results may be smaller than that for the aug-cc-pV5Z and aug-cc-pV6Z pair. Such nonsystematic behavior of non-CP-corrected correlation contributions has already been observed by Haklier *et al.*[38]

For both the aug-cc-pVXZ and aug′-cc-pVXZ series of basis sets an average of the three ΔE_{corr} CBS limits (ΔE_{avg}) is included in Table V along with conservative estimates of the error bars associated with the energy of each stationary point. These estimates were obtained from three distinct contributions. Data from the aug-cc-pVXZ series for structure #2 is used to illustrate the manner in which these error bars have been obtained.

(1) The uncertainty associated with the three MP2 CBS limits is estimated from the maximum deviation from the mean of the three CBS values for either the aug-cc-pVXZ or aug′-cc-pVXZ series of basis sets [e.g., $|0.67-(0.65+0.63+0.67)/3|=0.02$].
(2) For the CCSD(T) contribution, crude error bars can be obtained by averaging the change in $\delta E_{\mathrm{CCSD(T)}}$ between the two largest basis sets of each series [e.g., $(|0.07-0.09|+|0.08-0.07|)/2=0.02$].
(3) The contributions from higher order excitations are expected to be smaller than the average of the absolute value of $\delta E_{\mathrm{BD(TQ)}}$ for the double-ζ basis sets [e.g., $(|-0.01|+|-0.01|)/2=0.01$].

The reported error bars are simply the sum of these three contributions (e.g., $0.02+0.02+0.01=0.05$). Once again, we note that any inconsistencies in the tables are merely rounding errors. Each tabulated entry has been computed directly from the electronic energies (available in the supplementary material accompanying this paper[87]) and rounded to two decimal places. From the remainder of this section, it will become evident that contributions to the error bars from core correlation and the relativistic corrections are negligible.

As can be seen from Table V (and the corresponding supplementary material[87]), the agreement between the aug-cc-pVXZ and aug′-cc-pVXZ CBS limits is in general quite excellent. The only serious discrepancy (>0.05 kJ mol^{-1}) between the aug-cc-pVXZ and aug′-cc-pVXZ series occurs when the D_e is computed without a CP correction. For the aug-cc-pVXZ basis sets, the CP corrected and uncorrected CBS limits are, for all practical purposes, identical (20.84 vs 20.83, respectively). However, for the aug′-cc-pVXZ basis sets, the same values differ by 0.15 kJ mol^{-1} (20.81 versus 20.66). Such results are both encouraging and discouraging. For most applications, this small difference can be ignored in light of the substantial computational savings offered by the aug′-cc-pVXZ basis sets over their aug-cc-pVXZ counterparts (122 fewer basis functions for $X=6$). On the other hand, it is somewhat disappointing that the massive aug′-cc-pV6Z basis still suffers from a BSSE dissociation energy (D_e) correction that amounts to nearly 0.2 kJ mol^{-1}.

The effect of core correlation on the relative energies of these water dimer minima and transition states can be seen in

Downloaded 16 Feb 2003 to 130.207.165.29. Redistribution subject to AIP license or copyright, see http://ojps.aip.org/jcpo/jcpcr.jsp

the last column of Table VI. The complete list for all stationary points is provided in the supplementary material.[87] In most instances the effect is almost negligible (<0.03 kJ mol^{-1}). Only for two infinitely separated water monomers and the bifurcated structures does the contribution from core correlation approach or exceed 0.1 kJ mol^{-1}. The CBS limit of $\delta E_{+\mathrm{Core}}$ has essentially been reached since results obtained with the aug-cc-pCVQZ and aug-cc-pCV5Z basis sets differ by no more than 0.01 kJ mol^{-1}. Therefore, to estimate the contribution from core correlation we use the value of $\delta E_{+\mathrm{Core}}$ obtained with the aug-cc-pCV5Z basis set rather than employing an extrapolation scheme.

A relativistic correction to the electronic energy of each structure was obtained from CCSD(T) computations with the cc-pCVTZ basis set. Such corrections to the relative energies are essentially negligible and never exceed 0.01 kJ mol^{-1}. These results are displayed in Table VII along with other data summarizing the relative energies of the structures considered. In this table another estimate for ΔE_{avg} and the corresponding error bars is presented. The new values are obtained from all six CBS limits (three for the aug-cc-pVXZ series plus three from the aug$'$-cc-pVXZ series). For everything except the uncorrected D_e, the values of ΔE_{avg} reported in Table VII are almost identical to those in Table V (and in the corresponding table of the supplementary material[87]).

Also tabulated for comparison are the energies from the 1990 study by Smith *et al.*[41] as well as the barrier heights from several recently developed 6D IPESs (Refs. 42 and 43) for the water dimer. It is interesting to note that although the relative energies of the stationary points change by as much as 1.5 kJ mol^{-1} their order remains the same as in Ref. 41 (e.g., structure #8 is the highest). Comparison to the IPES results is far less straightforward. In fact, direct comparison of reported barrier heights to the relative energies of this study is dubious at best. The 6D surfaces are effective potentials and include an averaging of the zero-point effects from the six degrees of freedom not explicitly considered. It is not clear how the vibrationless energies reported here can be properly compared to the effective barrier heights from the empirically fit VRT(ASP-W) surface of Fellers *et al.*[42] or the *ab initio* symmetry-adapted perturbation theory (SAPT) based surfaces of Mas and co-workers.[43] As noted earlier, the lowest rotational transition state on the SAPT surfaces is planar and resembles structure #3. A stationary point of C_1 symmetry like structure #2 was not found. The present vibrational analysis at the CCSD(T) level with the TZ2P(f,d) +dif basis set supports the original characterization of the Hessian index for each stationary point.[41]

The potential confusion alluded to in the previous paragraph is further complicated by the highly anharmonic nature of the water dimer PES. To minimize the possibility of incorrect comparisons, the zero-point corrections to the best estimates of the relative energies (ΔE_e) are given here rather than in Table VII. After correcting ΔE_e for the unscaled, harmonic zero-point vibrational energy of each stationary point, we obtain values for ΔE_0^h of $+0.00$, $+0.56$, -0.45, $+1.45$, $+1.08$, $+0.43$, $+4.38$, $+9.81$, $+4.20$, $+5.66$, $+11.75$, and $+11.64$ kJ mol^{-1} for structures #1–#10, $D_0^{h,\mathrm{CP}}$, and D_0^h, respectively. One immediately notices that the harmonic approximation predicts that structure #3 (a second order saddle point) is 0.45 kJ mol^{-1} lower in energy than the global minimum (structure #1). Although these ZPVE corrected energies give some insight into the vibrational effects of this system, clearly they must be used judiciously.

IV. CONCLUSIONS

The present study has carried out an extensive *ab initio* quantum chemical study of ten stationary points on the water dimer PES. Full (12 degrees of freedom) geometry optimizations were carried out using the CCSD(T) method in conjunction with the TZ2P(f,d)+dif basis set. Harmonic vibrational frequencies were also computed at the same level. Some interesting qualitative conclusions about the nature of the $(H_2O)_2$ PES could be drawn from these preliminary calculations.

(1) The optimized structures reported here are generally quite similar to those obtained at the MP2 level with much smaller basis sets [6+31+G(d,p) and 6-311 +G(d,p)].[41]
(2) Only for bifurcated structures do connected triple excitations and a larger basis set lead to substantial geometrical changes.
(3) The Hessian index or number of imaginary vibrational frequencies of each stationary point is unchanged from the earlier study[41] despite fairly significant geometrical differences for some of the optimized structures.
(4) Structure #1 (nonplanar open C_s) is the only minimum.
(5) Structures #2 (open C_1), #4 (cyclic C_i), and #9 (nonplanar bifurcated C_{2v}) are transition states while the remaining stationary points are higher-order saddle points. Structure #2 does not appear to exist on two recently developed SAPT IPESs.[43] Instead, they find a transition state similar to structure #3 [a second-order saddle point at the CCSD(T)/TZ2P(f,d)+dif level].

After the structural and vibrational analysis, an exhaustive series of computations were performed to accurately characterize the relative energies of the ten stationary points. The main results are summarized in Table VII, but we reemphasize some of the more important results of the previous section.

(a) With the aug-cc-VXZ basis sets ($X=D,T,Q,5,6$), the relative electronic energies (including triple and quadruple excitations, core correlation, and even relativistic corrections) of ten stationary points on the water dimer PES have been converged to approximately ±0.3 kJ mol^{-1} or less (within the Born–Oppenheimer approximation).
(b) Although the relative energies reported here differ from the earlier MP2 values[41] by as much as 1.5 kJ mol^{-1}, the ordering from most stable to least stable remains unchanged.
(c) The maximum contributions from the treatment of electron correlation beyond the MP2 level is less than 0.7 kJ mol^{-1} (-0.7 kJ mol^{-1} for structure #8).

Downloaded 16 Feb 2003 to 130.207.165.29. Redistribution subject to AIP license or copyright, see http://ojps.aip.org/jcpo/jcpcr.jsp

(d) The maximum contribution from the core correlation is less than 0.2 kJ mol^{-1} (+0.2 kJ mol^{-1} for the CP corrected D_e).
(e) The maximum contribution from relativistic effects is less than 0.01 kJ mol^{-1} (−0.01 for the CP corrected D_e).
(f) While the CP corrected and uncorrected dissociation energies are essentially identical for the CBS limits from the aug-cc-pV*X*Z results, the aug′-cc-pV*X*Z series still suffers from a BSSE of nearly 0.2 kJ mol^{-1}.
(g) Utilizing both the CP corrected and uncorrected aug-cc-pV*X*Z data, an electronic dissociation energy of 21.0 ±0.3 kJ mol^{-1} for the water dimer is inferred.
(h) Our current best estimate agrees perfectly with the recent values obtained by Klopper and Lüthi[37] of $D_e = 21.1 \pm 0.3$ kJ mol^{-1} and by Klopper, van Duijneveldt–van de Rijdt, and van Duijneveldt[39] of $D_e = 21.0 \pm 0.2$ kJ mol^{-1} despite using rather different dimer geometries.

ACKNOWLEDGMENTS

This research has been supported financially by several sources including the ETH Zürich (including C4 and CSCS), the Schweizerischer Nationalfonds under Grant Nos. 20-50719.97 and 20.58985.99 and the National Science Foundation under Grant No. CHE-9827468. The authors thank Curt Janssen and Ida Nielsen for help with MPQC and Mike Colvin for providing computer time on ASCI Blue Pacific at Lawrence Livermore National Laboratory. Sandia is a multiprogram laboratory operated by Sandia Corporation, a Lockheed Martin Company, for the United States Department of Energy under Contract No. DE-AC04-94AL85000.

[1] *Water a Comprehensive Treatise: The Physics and Physical Chemistry of Water*, edited by F. Franks (Plenum, New York, 1972), Vol. 1.
[2] G. A. Jeffrey and W. Saenger, *Hydrogen Bonding in Biological Structures* (Springer-Verlag, Berlin, 1991).
[3] D. J. Wales, in *Encyclopedia of Computational Chemistry*, edited by P. v. R. Schleyer (Wiley, Chichester, 1998), Vol. 5, pp. 3183–3193.
[4] P. G. Hill, J. Phys. Chem. Ref. Data **19**, 1233 (1990).
[5] A. J. Stone, *The Theory of Intermolecular Forces* (Oxford University Press, Oxford, 1996).
[6] G. A. Jeffrey, *An Introduction to Hydrogen Bonding* (Oxford University Press, Oxford, 1997).
[7] S. Scheiner, *Hydrogen Bonding: A Theoretical Perspective* (Oxford University Press, Oxford, 1997).
[8] *Molecular Interactions from van der Waals to Strongly Bound Complexes*, edited by S. Scheiner (Wiley, Chichester, 1997).
[9] *Theoretical Treatments of Hydrogen Bonding*, edited by D. Hadži (Wiley, Chichester, 1997).
[10] A. Karpfen, in *Molecular Interactions*, edited by S. Scheiner (Wiley, New York, 1997).
[11] S. Scheiner, Annu. Rev. Phys. Chem. **45**, 23 (1994).
[12] M. A. Suhm and D. J. Nesbitt, Chem. Soc. Rev. **24**, 45 (1995).
[13] Z. Bačić and R. E. Miller, J. Phys. Chem. **100**, 12945 (1996).
[14] G. C. Pimentel and A. L. McClellan, *The Hydrogen Bond* (Freeman, San Francisco, 1960).
[15] S. N. Vinogradov and R. H. Linnell, *Hydrogen Bonding* (Van Nostrand Reinhold, New York, 1971).
[16] R. C. Cohen and R. J. Saykally, Annu. Rev. Phys. Chem. **42**, 369 (1991).
[17] M. Quack and M. A. Suhm, in *Conceptual Perspectives in Quantum Chemistry*, edited by J.-L. Calais and E. S. Kryachko (Kluwer, Dordrecht, 1997), Vol. III.
[18] M. A. Suhm, Ber. Bunsenges. Phys. Chem. **99**, 1159 (1995).
[19] M. Kofranek, H. Lischka, and A. Karpfen, Chem. Phys. **121**, 137 (1988).
[20] M. Quack and M. A. Suhm, J. Chem. Phys. **95**, 28 (1991).
[21] W. Klopper, M. Quack, and M. A. Suhm, Chem. Phys. Lett. **261**, 35 (1996).
[22] W. Klopper, M. Quack, and M. A. Suhm, J. Chem. Phys. **108**, 10096 (1998).
[23] M. Quack, J. Stohner, and M. A. Suhm, J. Mol. Struct. **294**, 33 (1993).
[24] M. Quack, J. Stohner, and M. A. Suhm, J. Mol. Struct. (in press).
[25] K. von Puttkamer and M. Quack, Chem. Phys. **139**, 31 (1989).
[26] L. Oudejans and R. E. Miller, J. Chem. Phys. **113**, 971 (2000).
[27] Z. Bačić and Y. Qui, in *Advances in Molecular Vibrations and Collision Dynamics*, edited by J. Bowman and Z. Bačić (JAI, Greenwich, 1998), Vol. III.
[28] D. H. Zhang, Q. Wu, J. Z. H. Zhang, M. von Dirke, and Z. Bačić, J. Chem. Phys. **102**, 2315 (1995).
[29] O. Matsuoka, E. Clementi, and M. Yoshimine, J. Chem. Phys. **64**, 1351 (1976).
[30] D. R. Yarkony, S. V. O'Neil, H. F. Schaefer, C. P. Baskin, and C. F. Bender, J. Chem. Phys. **60**, 885 (1974).
[31] C. Millot and A. J. Stone, Mol. Phys. **77**, 439 (1992).
[32] K. Morokuma and L. Pedersen, J. Chem. Phys. **48**, 3275 (1968).
[33] K. Morokuma and J. R. Winick, J. Chem. Phys. **52**, 1301 (1970).
[34] P. A. Kollman and L. C. Allen, J. Chem. Phys. **52**, 5085 (1970).
[35] G. H. F. Diercksen and W. P. Kraemer, Chem. Phys. Lett. **6**, 419 (1970).
[36] W. Klopper, M. Quack, and M. A. Suhm, Mol. Phys. **94**, 105 (1998).
[37] W. Klopper and H. P. Lüthi, Mol. Phys. **96**, 559 (1999).
[38] A. Halkier, W. Klopper, T. Helgaker, P. Jørgensen, and P. R. Taylor, J. Chem. Phys. **111**, 9157 (1999).
[39] W. Klopper, J. G. C. M. van Duijneveldt–van de Rijdt, and F. B. van Duijneveldt, Phys. Chem. Chem. Phys. **22**, 2227 (2000).
[40] E. M. Mas and K. Szalewicz, J. Chem. Phys. **104**, 7606 (1996).
[41] B. J. Smith, D. J. Swanton, J. A. Pople, H. F. Schaefer, and L. Radom, J. Chem. Phys. **92**, 1240 (1990).
[42] R. S. Fellers, C. L. L. B. Braly, M. G. Brown, and R. J. Saykally, Science **284**, 945 (1999).
[43] E. M. Mas, R. Bukowski, K. Szalewicz, G. C. Groenenboom, P. E. S. Wormer, and A. van der Avoird, J. Chem. Phys. **113**, 6687 (2000).
[44] A. L. L. East and W. D. Allen, J. Chem. Phys. **99**, 4638 (1993).
[45] W. D. Allen, A. L. L. East, and A. G. Császár, in *Structures and Conformations of Non-Rigid Molecules*, edited by J. Laane, M. Dakkouri, B. van der Veken, and H. Oberhammer (Kluwer, Dordrecht, 1993).
[46] B. D. Wladkowski, W. D. Allen, and J. I. Brauman, J. Am. Chem. Soc. **98**, 13532 (1994).
[47] S. J. Klippenstein, A. L. L. East, and W. D. Allen, J. Chem. Phys. **105**, 118 (1996).
[48] N. L. Allinger, J. T. Fermann, W. D. Allen, and H. F. Schaefer, J. Chem. Phys. **106**, 5143 (1997).
[49] A. G. Császár, W. D. Allen, and H. F. Schaefer, J. Chem. Phys. **108**, 9751 (1998).
[50] G. Tarczay, A. G. Császár, W. Klopper, V. Szalay, W. D. Allen, and H. F. Schaefer, J. Chem. Phys. **110**, 11971 (1999).
[51] G. S. Tschumper, Ph.D. thesis, University of Georgia, Athens, Georgia, 1999.
[52] R. J. Bartlett, Annu. Rev. Phys. Chem. **32**, 359 (1981).
[53] G. D. Purvis and R. J. Bartlett, J. Chem. Phys. **76**, 1910 (1982).
[54] G. E. Scuseria, A. C. Scheiner, T. J. Lee, J. E. Rice, and H. F. Schaefer, J. Chem. Phys. **86**, 2881 (1987).
[55] G. E. Scuseria, C. L. Janssen, and H. F. Schaefer, J. Chem. Phys. **89**, 7382 (1988).
[56] K. Raghavachari, G. W. Trucks, J. A. Pople, and M. Head-Gordon, Chem. Phys. Lett. **157**, 479 (1989).
[57] T. H. Dunning, J. Chem. Phys. **55**, 716 (1971).
[58] S. Huzinaga, J. Chem. Phys. **42**, 1293 (1965).
[59] M. J. Frisch, J. A. Pople, and J. S. Binkley, J. Chem. Phys. **80**, 3265 (1984).
[60] T. J. Lee and H. F. Schaefer, J. Chem. Phys. **83**, 1784 (1985).
[61] P. Pulay, Mol. Phys. **17**, 197 (1969).
[62] J. F. Stanton, J. Gauss, W. J. Lauderdale, J. D. Watts, and R. J. Bartlett, ACES II. The package also contains modified versions of the MOLECULE Gaussian integral program of J. Almlöf and P. R. Taylor, the ABACUS integral derivative program written by T. U. Helgaker, H. J. Aa. Jensen, P. Jørgensen, and P. R. Taylor, and the PROPS property evaluation integral code of P. R. Taylor.
[63] G. E. Scuseria, J. Chem. Phys. **94**, 442 (1991).

Downloaded 16 Feb 2003 to 130.207.165.29. Redistribution subject to AIP license or copyright, see http://ojps.aip.org/jcpo/jcpcr.jsp

[64] G. S. Tschumper, M. D. Kelty, and H. F. Schaefer, Mol. Phys. **96**, 493 (1999).
[65] R. A. Kendall, T. H. Dunning, and R. J. Harrison, J. Chem. Phys. **96**, 6796 (1992).
[66] T. H. Dunning, J. Chem. Phys. **90**, 1007 (1989).
[67] C. Møller and M. S. Plesset, Phys. Rev. **46**, 618 (1934).
[68] J. A. Pople, J. S. Binkley, and R. Seeger, Int. J. Quantum Chem., Symp. **10**, 1 (1976).
[69] N. C. Handy, J. A. Pople, M. Head-Gordon, K. Raghavachari, and G. W. Trucks, Chem. Phys. Lett. **164**, 185 (1989).
[70] K. Raghavachari, J. A. Pople, E. S. Replogle, and M. Head-Gordon, J. Phys. Chem. **94**, 5579 (1990).
[71] C. J. Janssen, E. T. Seidl, and M. E. Colvin, in *Parallel Computing in Computational Chemistry, ACS Symposium, Series 592*, edited by T. G. Mattson (American Chemical Society, Washington, DC, 1995), p. 47.
[72] I. M. B. Nielsen and E. T. Seidl, J. Comput. Chem. **16**, 1301 (1995).
[73] I. M. B. Nielsen, Chem. Phys. Lett. **255**, 210 (1996).
[74] M. J. Frisch, G. W. Trucks, H. B. Schlegel *et al.*, GAUSSIAN 94, Revision C.3, Gaussian, Inc., Pittsburgh, PA, 1995.
[75] D. Feller, J. Chem. Phys. **96**, 6104 (1992).
[76] D. Feller, J. Chem. Phys. **98**, 7059 (1993).
[77] T. Helgaker, W. Klopper, H. Koch, and J. Noga, J. Chem. Phys. **106**, 9639 (1996).
[78] W. Kutzelnigg and W. Klopper, J. Chem. Phys. **94**, 1985 (1991).
[79] W. Klopper, Chem. Phys. Lett. **186**, 583 (1991).
[80] E. F. Valeev and H. F. Schaefer, J. Chem. Phys. **113**, 3990 (2000).
[81] T. D. Crawford, C. D. Sherrill, E. F. Valeev *et al.*, PSI 3.0, PSITECH, Inc., Watkinsville, Georgia, 2000.
[82] W. Klopper and J. Noga, J. Chem. Phys. **103**, 6127 (1995).
[83] D. E. Woon and T. H. Dunning, J. Chem. Phys. **103**, 4572 (1995).
[84] B. Liu and A. D. McLean, J. Chem. Phys. **59**, 4557 (1973).
[85] H. B. Jansen and P. Ros, Chem. Phys. Lett. **3**, 140 (1969).
[86] S. F. Boys and F. Bernardi, Mol. Phys. **19**, 553 (1970).
[87] See EPAPS Document No. E-JCPSA6-115-307141 for seven tables containing Cartesian coordinates, harmonic vibrational frequencies, infrared intensities, electronic energies, relativistic corrections, and focal point analyses for the ten stationary points of $(H_2O)_2$ as well as H_2O. This document may be retrieved via the EPAPS homepage (http://www.aip.org/pubservs/epaps.html) or from ftp.aip.org in the directory /epaps/. See the EPAPS homepage for more information.
[88] P. Hobza, O. Bludský, and S. Sunhai, Phys. Chem. Chem. Phys. **1**, 3073 (1999).
[89] A. Halkier, H. Koch, P. Jørgensen, O. Christiansen, I. M. B. Nielsen, and T. Helgaker, Theor. Chim. Acta **97**, 150 (1997).

Downloaded 16 Feb 2003 to 130.207.165.29. Redistribution subject to AIP license or copyright, see http://ojps.aip.org/jcpo/jcpcr.jsp
