# Supplementary material for: Computational chemistry methods based on MNDO and tools for improving their accuracy
Source: J Mol Model. 2026 Mar 13;32(4):98. doi: 10.1007/s00894-026-06653-3 (PMC12987868; doi:10.1007/s00894-026-06653-3)
Supplement: Supplementary file 1 — ESM1 (ZIP 34.0 MB) [file 894_2026_6653_MOESM1_ESM.zip › jsmol/j2s/J/bspt/package.html]

Provides Binary Space Partitioning Functionality to spacially
separate data structures in n-dimensions

For some useful background info, search the web for "bsp tree faq".
