# Supplementary material for: Computational chemistry methods based on MNDO and tools for improving their accuracy
Source: J Mol Model. 2026 Mar 13;32(4):98. doi: 10.1007/s00894-026-06653-3 (PMC12987868; doi:10.1007/s00894-026-06653-3)
Supplement: Supplementary file 1 — ESM1 (ZIP 34.0 MB) [file 894_2026_6653_MOESM1_ESM.zip › jsmol/j2s/J/g3d/package.html]

Provides a software implementation of complete 3D graphics engine
for rendering molecules and associated shapes.
basic shapes.

This package implements all graphics primitives used by Jmol. The
graphics engine does not use Java3D or OpenGL. It requires no specialized
graphics hardware. This allows Jmol and the JmolApplet to run on any
machine that has a JVM.

It is a software implementation of a graphics z-buffer. An int[] called
pbuf (for pixel buffer) is used to hold ARGB values for pixels. A short[]
called zbuf holds the z-depth value.

Shape primitives are provided for drawing spheres, cylinders, triangles, etc.
Shading primitives provide lighting/shading support

## Related Documentation

Makes use of javax.vecmath classes.
