# Supplementary material for: Computational chemistry methods based on MNDO and tools for improving their accuracy
Source: J Mol Model. 2026 Mar 13;32(4):98. doi: 10.1007/s00894-026-06653-3 (PMC12987868; doi:10.1007/s00894-026-06653-3)
Supplement: Supplementary file 1 — ESM1 (ZIP 34.0 MB) [file 894_2026_6653_MOESM1_ESM.zip › jsmol/j2s/JU/colorschemes/_schemes.htm]

### batlow

### cividis

### inferno

### kry

### magma

### plasma

### thermal

### turbo

### viridis
